# Pyropheophorbide-a/(001) TiO_2_ Nanocomposites with Enhanced Charge Separation and O_2_ Adsorption for High-Efficiency Visible-Light Degradation of Ametryn

**DOI:** 10.3390/molecules27175576

**Published:** 2022-08-30

**Authors:** Songtao Liu, Rui Yan, Muhammad Humayun, Huanli Zhang, Yang Qu, Yingxue Jin

**Affiliations:** 1 Key Laboratory for Photonic and Electronic Bandgap Materials, Ministry of Education, College of Chemistry & Chemical Engineering, Harbin Normal University, Harbin 150025, China; 2 Wuhan National Laboratory for Optoelectronics, Optics Valley Laboratory, School of Optical & Electronics Information, Huazhong University of Science & Technology, Wuhan 430074, China; 3 Key Laboratory of Functional Inorganic Materials Chemistry (Ministry of Education), School of Chemistry and Materials Science, International Joint Research Center and Lab for Catalytic Technology, Heilongjiang University, Harbin 150080, China; 4 Key Laboratory of Molecular Cytogenetics and Genetic Breeding of Heilongjiang Province, College of Life Science and Technology, Harbin Normal University, Harbin 150025, China

**Keywords:** Ppa, 001-facet-exposed TiO_2_, charge separation, visible-light photocatalysis, Ametryn

## Abstract

It is highly desired to enhance charge separation and O_2_ adsorption of the pyropheophorbide-a (Ppa) to promote visible-light activity and stability. Herein, Ppa modified 001-facet-exposed TiO_2_ nanosheets (Ppa/001T) nanocomposites with different weight ratios were fabricated via the self-assembly approach by OH induced. Compared with the bare Ppa, the 8% amount optimized 8Ppa/001T sample displayed 41-fold enhanced activity for degradation of Ametryn (AME) under visible-light irradiation. The promoted photoactivities could be attributed to the accelerated charge carrier’s separation by coupling TiO_2_ as thermodynamic platform for accepting the photoelectrons with high energy from Ppa and the promoted O_2_ adsorption because of the residual fluoride on TiO_2_. As for this, a distinctive two radicals (•O_2_^−^ and •OH) involved pathway of AME degradation is carried out, which is different from the radical pathway dominated by •O_2_^−^ for the bare Ppa. This work is of utmost importance since it gives us detailed information regarding the charge carrier’s separation and the impact of the radical pathway that will pave a new approach toward the design of high activity visible-light driven photocatalysts.

## 1. Introduction

Ametryn (AME) as a triazine herbicide is used worldwide to improve the yield of agricultural products [1]. It is classified as a Class-III herbicide by the United States Environmental Protection Agency (EPA) and banned for agricultural use in the European Union (EU) in 2002 [2,3,4] due to its highly stable nature, high solubility in water, and weak adsorption behavior [5,6], causing cancerous tumors after long exposure [7,8], which has been designated as an endocrine disrupting compound. However, it is still present in water and soil samples, and its concentration can even reach 3.4 mg L^−1^ [9]. Thus, effective removal of AME has received worldwide scientific attention due to health and eco-friendly safety alarms.

Photocatalysis has been recognized as a promising advanced oxidation technology for pollutant elimination owing to its effective, mild, green, and environmentally-friendly merits [10,11]. Thus, it is a viable strategy for AME degradation, relying on the photocatalysts to generate highly reactive oxygen species (ROS) under light radiation to achieve oxidized intermediate products [12]. Yet, the development of novel and efficient photocatalysts still remains a challenge. In recent years, it has been found that some dye molecules can be used as photocatalysts owing to their higher molar absorption coefficient and wide range light absorption spectrum. Ppa is a natural chlorophyll derivative with a broad absorption wavelength and has many advantages such as the ease of synthesis due to the availability of abundant precursors, clear molecular structure and elemental composition, and environmentally-friendly merits [13,14]. Nevertheless, the photocatalytic performance of Ppa is still insufficient mainly due to the poor stability and ease of self-degradation and further lack of oxygen activation centers for ROS production. Therefore, it is of utmost importance to improve the photocatalytic activity of Ppa for AME degradation synergistically by increasing its stability, inhibiting self-oxidation, and generating ROS oxygen active centers.

The self-oxidation and poor stability of Ppa is closely related to the rapid charge recombination rate. Research shows that the construction of heterostructure composites is an innovative strategy for enhancing the charge carrier’s separation among the widely used modification approaches [15]. Therefore, the selection of suitable materials to build heterojunctions is the key factor, and the selected materials need to meet the following conditions: (i) appropriate position of the conduction band; (ii) surface structure match; and (iii) oxygen adsorption capacity.

Anatase TiO_2_ nanosheets with exposed (001) facets is a suitable material that can be employed as a proper energy platform for Ppa, since it is cost-effective, exhibits high chemical stability, and has an appropriate conduction band bottom level. Thus, it can facilitate the maintenance of electrons at a high thermodynamic level and can be advantageous for photocatalysis and especially for the activation of oxygen [16,17,18]. Fortunately, it has been found that 001-facet-exposed TiO_2_ with 2D structure can be coupled with Ppa through effective interfacial contact [19], thereby promoting charge transfer and separation. In addition, its surface fluorine residue could significantly improve the adsorption of oxygen in order to promote the electrons capture by the surface adsorbed O_2_, leading to the enhanced charge carrier’s separation and improved photoactivities [20]. Therefore, the combination of these properties of 001-facet-exposed TiO_2_ is expected to solve the problem of Ppa, thereby promoting the photocatalyst performance for degrading AME. Noticeably, related works have seldom been reported to date.

Based on the above considerations, herein, we fabricated Ppa/(001)TiO_2_ (denoted as Ppa/001T) photocatalysts by a simple self-assemble approach via OH induced. The promoted photoactivity could be attributed to the improved charge carrier’s separation by coupling 001T as the thermodynamic platform for accepting the high energy photoinduced electrons of Ppa and to the promoted O_2_ adsorption because of the residual fluoride on 001T. As for this, a distinctive two radicals (•O_2_^−^ and •OH) involved pathway of AME degradation is proposed, which is different from the radical pathway dominated by •O_2_^−^ for the bare Ppa. This work will provide new insights into the degradation mechanism of Ppa and will help researchers design high performance visible-light driven photocatalysts.

## 2. Results

### 2.1. Structural Characterization

**Scheme 1:**
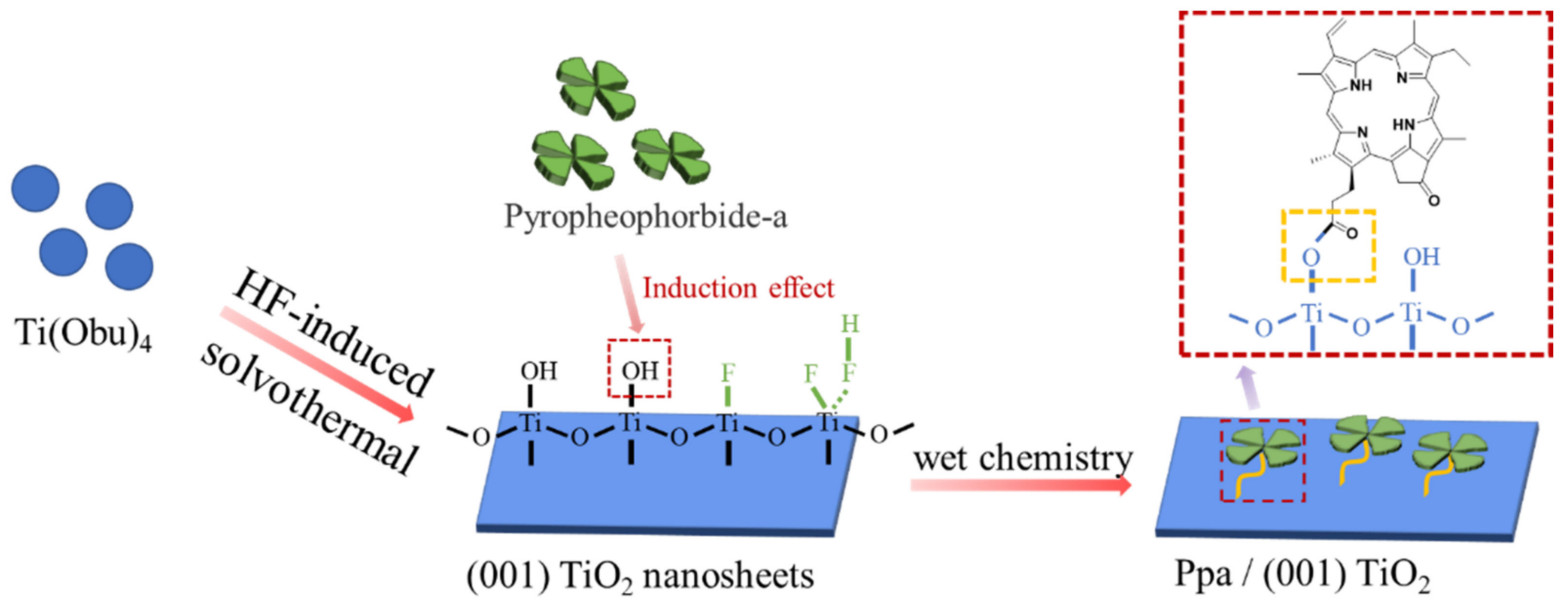
Synthetic illustration of the Ppa/001T heterojunction.

The synthetic process of dimension-matched Ppa/001T heterojunction is shown in Scheme 1. The Ppa/001T heterojunctions were fabricated using a wet chemical method through a simple self-assemble approach via OH induced. It can be seen from Scheme 1 that the Ppa has enriched hydrophilic carboxyl groups, which is beneficial for the photocatalytic reactions over the designed composite. Worth noting, Ppa can tightly anchor onto the surface of 001T due to the interaction of the carbonyl group of Ppa and the Ti-O group of the 001T. Thus, Ppa can uniformly anchor onto the surface of 001T, forming a Ppa/001T heterojunction, which can effectively inhibit the photodegradation of Ppa via the fast charge separation and transfer [21].

**Figure 1:**
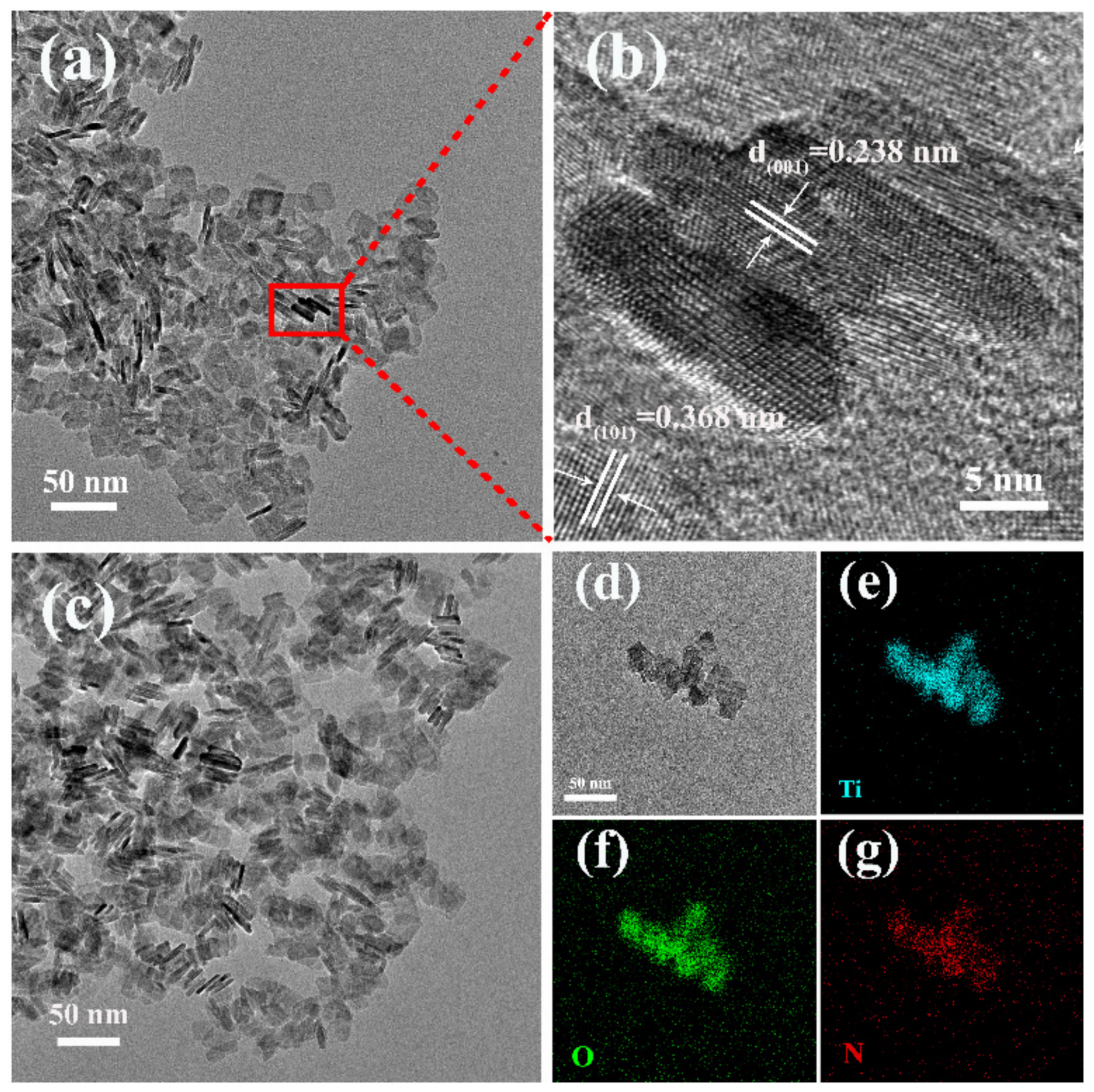
(**a**) TEM and (**b**) HRTEM images of 001T; (**c**) TEM of 8Ppa/001T; (**d**) TEM and (**e**–**g**) EDS mapping images of the corresponding Ti, O, and N elements of 8Ppa/001T.

Transmission electron microscopy (TEM) and thigh-resolution (HR) TEM studies were performed to reveal the structural composition of 001T, Ppa/001T, and spatial distribution of Ppa on the 001T matrix. As revealed in Figure 1a, the 001T nanoparticles shape is quadrate sheets with an average of 20 nm size. Two inter planar spacing of 0.238 and 0.368 nm is clearly observed in the HRTEM image as provided in Figure 1b, usually accredited to the (001) and (101) planes of TiO_2_, respectively [19,22]. There is no perceptible change after the introduction of Ppa (Figure 1c). The EDS mapping images of the 8Ppa/001T heterojunction reveals the even distribution of Ti, O, and N across the nanosheets, which clearly demonstrate that Ppa is uniformly distributed on the surface of 001T (Figure 1d–g).

To further study the structural properties of Ppa, 001T, and xPpa/001T photocatalysts, the X-ray diffractometer (XRD), diffuse reflectance spectra (DRS), Fourier-transform infrared spectrometer (FT-IR), and Raman spectra characterizations were performed. The XRD patterns of Ppa, 001T, and xPpa/001T composites are shown in Figure S1. The XRD patterns of bare 001T can be indexed to the anatase phase since no other crystal phases such as brookite and rutile can be observed. Further, the phase composition of 001T remain unaffected after coupling with Ppa, owing to the low content and high dispersion of Ppa. Thus, the Ppa modification on the 001T surface has little effect on the anatase 001 crystal structure.

**Figure 2:**
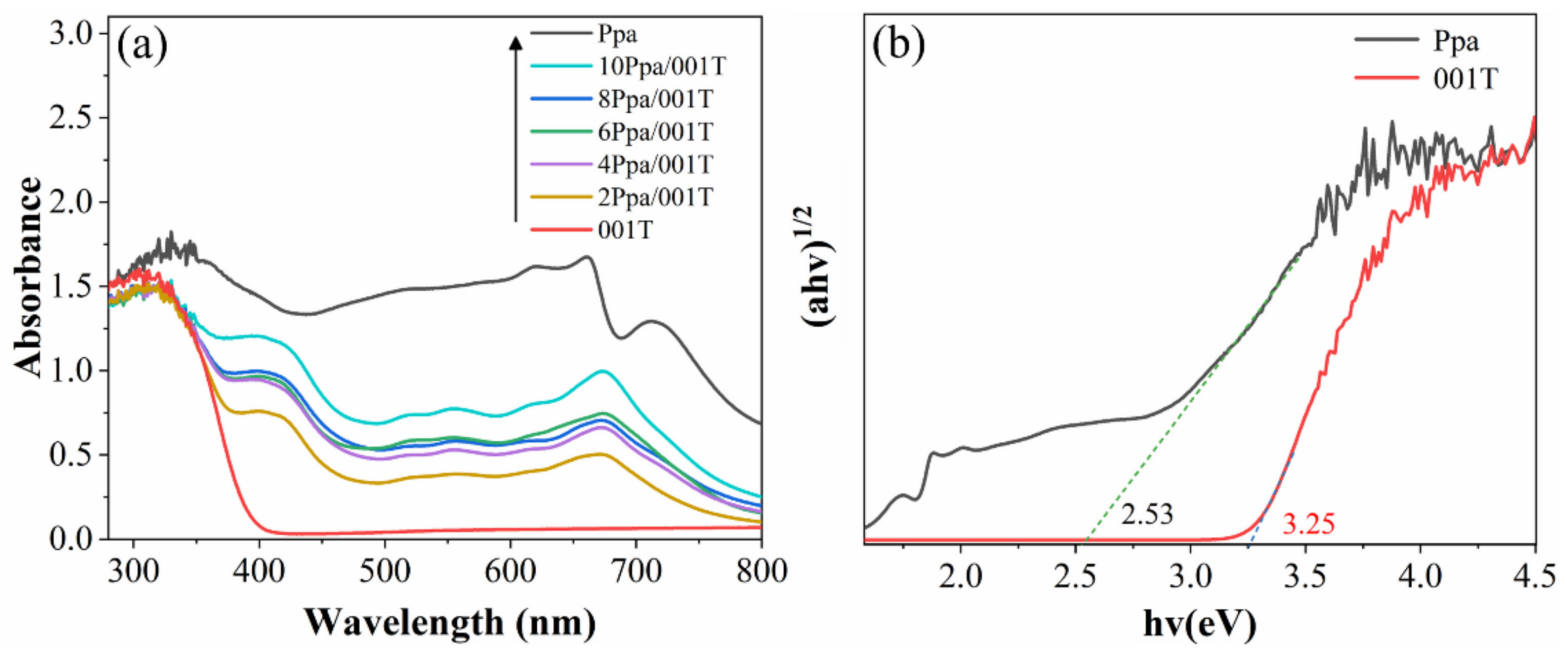
(**a**) DRS spectra of Ppa, 001T, xPpa/001T; (**b**) estimated band gap curves of Ppa and 001T.

The UV-visible DRS spectra in Figure 2a clearly reveals the characteristic absorption peaks of Ppa in the Ppa/001T composites and their peaks intensity slightly enhanced by increasing the percentage content. Compared to the absorption band edge of bare 001T, the xPpa/001T sample showed a strong peak at 412 nm corresponding to the Soret-bands, and four weak peaks at 507, 538, 610, and 667 nm, corresponding to Q-bands of Ppa [23]. Further, the absorption edges of xPpa/001T composites are significantly red-shifted, which can be attributed to the interactions or noncovalent bonding between Ppa and 001T. Thus, the UV-vis DRS spectra results prove that Ppa is successfully modified onto the surface of 001T. The band gaps (Eg) of the bare 001T and Ppa photocatalysts were predicted from Tauc plots derived from the Kubelka–Munk function and are shown in Figure 2b. The Ppa sample revealed a narrow band gap of 2.53 eV compared to that of the 001T sample (i.e., 3.25 eV).

**Figure 3:**
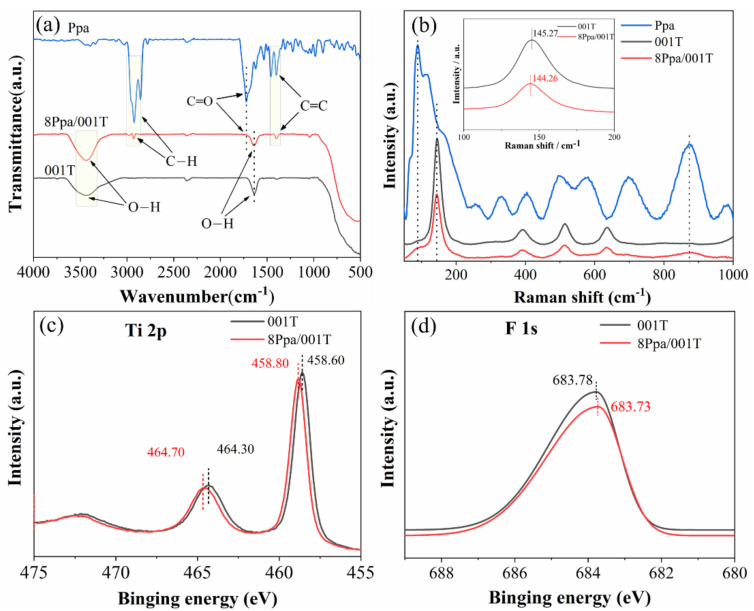
FT-IR (**a**) and Raman spectra (**b**) of Ppa, 001T, and 8Ppa/001T; and XPS spectra for Ti 2p of 001T and 8Ppa/001T (**c**) and for F1s of 001T and 8Ppa/001T (**d**).

To investigate the interfacial bonding mode of Ppa and 001T photocatalysts, FT-IR spectra analysis was conducted as revealed in Figure 3a. The broad peak ranging from 1000 to 470 cm^−1^ could be assigned to characteristic stretching vibrations of the Ti-O bond. The peaks at 1625 cm^−1^ and 3412 cm^−1^ could be accredited to the O-H bending and stretching vibration mode of the surface hydroxyl groups of the bare 001T. Noteworthy, the characteristic peaks of Ppa at 2968, 2921, 1726 cm^−1^, 1608, and 1548 cm^−1^ are detected in 8Ppa/001T samples, indicating the successful heterojunction of Ppa and 001T [22]. Interestingly, compared with 001T and bare Ppa, the stretching vibration peak of 1625 cm^−1^ in 8ppa/001T became weaker, and the C=O stretching vibration absorption peak at 1726 cm^−1^ slightly shifted in the direction of lower frequency, thus leading to a phase coincidence with the peak at 1625 cm^−1^, indicating that Ppa might chemisorbed onto the surface of 001T via the Ti-O-C=O bond, which is useful for the electron transfer between Ppa and the Ti (3d) orbital of 001T.

Raman technique is highly sensitive for detection of the changes in the lattice symmetry of various compounds. As shown in Figure 3b, the Raman spectrum peak at 394 cm^−1^ belongs to the symmetric-bending vibration, the peak at 514 cm^−1^ attributes to the antisymmetric-bending vibration, and the one at 144, 636 cm^−1^ is accredited to the symmetric stretching vibration of O-Ti-O in 001T [24]. Notably, the peak at 144 cm^−1^ of Ppa is blue-shifted, which might be due to the connection between carbonyl and Ti-O functionalities to form the Ti-O-C=O structure.

To further explore the change in surface chemical composition and the interaction between Ppa and 001T, X-ray photoelectron spectroscopy (XPS) spectra were measured. The Ti (2p) XPS spectra of 001T and 8Ppa/001T samples are revealed in Figure 3c. In the XPS spectrum of 001T, the peaks at binding energy values of 458.8 and 464.7 eV can be attributed to the Ti 2p3/2 and Ti 2p1/2 orbitals splitting of Ti 2p [25]. The Ti 2p binding energy peaks of 8Ppa/001T are slightly red-shifted compared to that of the 001T, indicating the electronic interaction between 001T and Ppa components. As revealed in Figure 3d, the binding energy peak of F 1s is centered at 684.0 eV, demonstrating the existence of Ti-F bonds on the surface of 001T [26]. Interestingly, the F 1 s binding energy peak also appeared in the 8Ppa/001T composite. The full, O1s, C1s, and N1s XPS spectra of bare Ppa and 8Ppa /001T are shown in Figure S2.

### 2.2. Photogenerated Charge Separation

**Figure 4:**
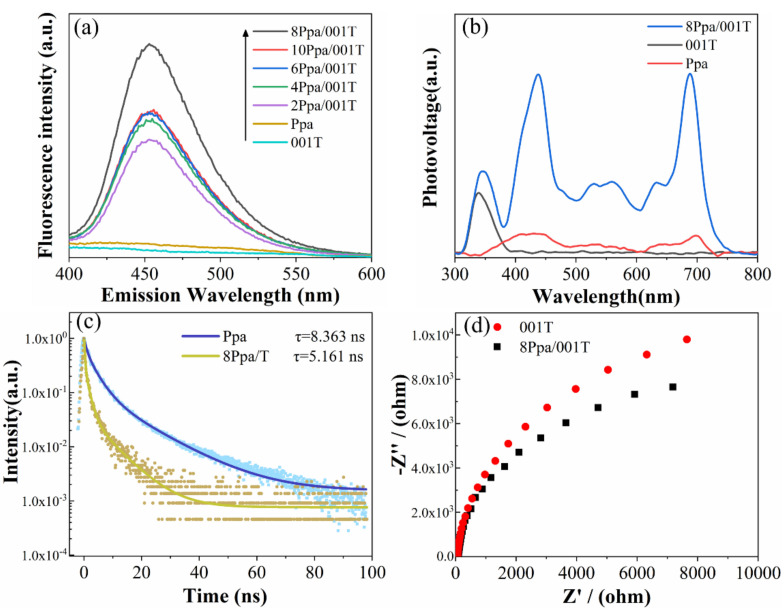
(**a**) FS spectra of the •OH radical over the 001T, Ppa, and xPpa/001T samples; (**b**) SS-SPS responses and (**c**) TR-PL responses of the Ppa and 8Ppa/001T; (**d**) Electrochemical impedance (EIS) spectra of the 001T and 8Ppa/001T samples.

The coumarin fluorescent technique was used to estimate the produced hydroxyl radical (•OH) amount. This vital method is employed to investigate the charge carrier’s separation, since the larger the •OH amount, the more efficient the charge carrier’s separation [27]. As shown in Figure 4a, the 001T and Ppa samples produced a very small amount of •OH as confirmed by its weak emission peak. However, after coupling 001T, the •OH generation over the Ppa sample is remarkably improved. Worth noting, by increasing the content of Ppa, the •OH-related emission peak became stronger and the highest amount was detected for the 8Ppa/001T sample. A further increase in the Ppa content strongly inhibited the amount of •OH, which might be due to the fact that the excess amount of Ppa leads to the structural agglomeration, demonstrating that it is highly beneficial for •OH production over the 001T to modify an appropriate amount of Ppa. To confirm the •OH test results, surface photovoltage spectra (SS-SPS) responses were measured as revealed in Figure 4b. The SS-SPS is a highly sensitive technique employed for investigation of the photophysical behavior of photo-induced charges in semiconductors and the SPS signals originate from charges separation via the diffusion phenomenon [28]. Clearly, the 001T sample reveals a narrow SPS peak centering at 340 nm. However, the Ppa sample revealed a broad SPS signal in the range of 300 to 800 nm. Worth noting, the SPS signal of the 8Ppa/001T sample is stronger in comparison to the bare 001T and Ppa samples, confirming enhanced charge separation in the fabricated composite. The result of SS-SPS is consistent with that of the •OH test. The enhanced charge separation is further confirmed via the time-resolved photoluminescence technique. The obtained time-resolved photoluminescence spectra (TR-PL) of the samples and the fitted parameters are revealed in Figure 4c and Table S2, respectively. As it is obvious, the fast decay component of Ppa and 8Ppa/001T samples revealed slower decay on the nanosecond time scale with average lifetimes “τ” of 8.363 and 5.161 ns. The results of the shortened fluorescence lifetime revealed that the electrons of Ppa are effectively transferred to 001T after compounding, and promoted charge transfer. Figure 4d reveals the electrochemical-impedance spectra (EIS) of the samples. The semicircle radius of the 8Ppa/001T is smaller than that of the bare 001T, indicating the fact that the 001T platform constructed for Ppa could remarkably reduce the charge transfer resistance, and well consistent with the SPS and TR-PL results. As revealed in Figure S3, significant enhancement in the photocurrent density-related I-V curves of the 8Ppa/001T sample is observed in comparison to that of the bare 001T and Ppa samples. This further strengthened the evidence of enhanced charge separation in the composite due to the interfacial charge transfer.

### 2.3. Photocatalytic Performance

**Figure 5:**
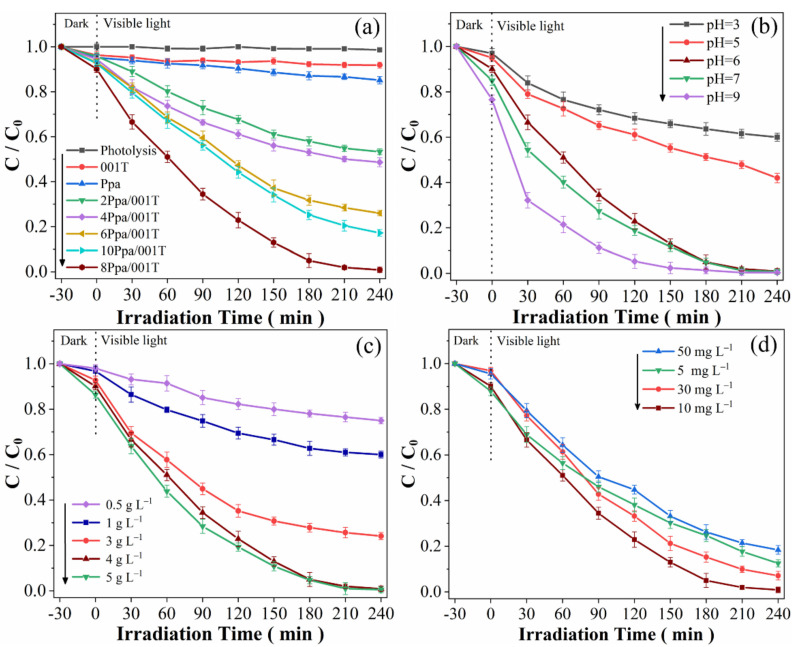
(**a**) Photocatalytic degradation of AME over the 001T, Ppa, and xPpa/001T samples under visible-light irradiation for 4 h; (**b**) Effect of the pH on the degradation of 10 mg L^−1^ AME pollutant over the 8Ppa/001T catalyst (4 g L^−1^); (**c**) Effect of the change in content of the 8Ppa/001T catalyst on the degradation of 10 mg L^−1^ AME at pH 6; (**d**) Effect of the solution concentration on the degradation rate of AME over the 8Ppa/001T catalyst at pH 6 (catalyst content = 4 g L^−1^).

The photocatalytic activity of the 001T, Ppa, and xPpa/001T samples was evaluated by the target pollutant (AME) degradation under visible-light (λ > 420 nm) irradiation for 4 h. Prior to the light irradiation, a control experiment (blank) was conducted without the addition of photocatalyst. Then, each of the photocatalyst was dispersed in an appropriate volume of AME solution and kept under stirring in the dark for 30 min to accomplish the adsorption–desorption equilibrium. As seen in Figure 5a, the composite samples reveal better adsorption for the AME pollutant in comparison to those of the bare 001T and Ppa samples. About 10% adsorption is achieved for the optimized 8Ppa/001T sample. This reveals that the adsorption also contributes to the total degradation. After the adsorption reaction, the system was irradiated under visible light for 4 h while stirring continuously. The 001T has almost zero degradation performance because it is inactive under visible-light irradiation. Besides, the bare Ppa also exhibits very low degradation performance (i.e., ~10%). As it is obvious, after coupling 001T, the degradation performance of the xPpa/001T photocatalyst is remarkably enhanced. As the content of Ppa in the composites increases, the percentage degradation of AME also increases. As expected, the 8Ppa/001T revealed the highest performance by fully degrading the AME pollutant. Compared with bare Ppa, 8Ppa/001T showed a 41-fold higher degradation rate, as shown in Figure S4a.

To further investigate the pH effect on the visible-light catalytic degradation of AME (10 mg L^−1^) over the optimized 8Ppa/001T sample, different pH values were used. The change in pH slightly influenced the adsorption of the photocatalyst (Figure 5b), while the photocatalytic degradation rate of AME favored a highly basic trend, which may be related to the pKa (3.71) value of AME. Based on the strong acidity of AME, the alkaline condition promoted the photocatalytic degradation of AME to some extent, showing a little enhancement in the kinetic curve. The effect of 8Ppa/001T content over the degradation rate of AME (10 mg L^−1^) was evaluated at pH 6, considering the later environmental pollution problem. As seen in Figure 5c, the degradation rate of AME is significantly improved by increasing the content of the 8Ppa/001T catalyst, especially for the addition of 4 g L^−1^ content. To verify the potential homogeneous reaction, the effect of the AME solution concentration on the degradation rate over the 8Ppa/001T catalyst (content = 4 g L^−1^) at pH 6 was evaluated. As shown in Figure 5d, the degradation rate tends to increase to some extent as the AME solution concentration decreases (50–10 mg L^−1^). However, as the concentration was further reduced, the degradation rate showed a decreasing trend instead. This might be due to the fact that the effective contact between the catalyst and the pollutant is very small, which can influence the degradation rate. For practical applications, we also investigated the effect of inland lake water and local tap water (used for the preparation of the AME solution, 10 mg L^−1^) on the degradation rate of AME over the 8Ppa/001T catalyst (content = 4 g L^−1^) at pH 6. As seen in Figure S4b, about 60 and 80% of AME is eliminated from the inland lake water and tap water, respectively, in the presence of the 8Ppa/001T catalyst. This further confirms the practical applicability of the catalyst.

### 2.4. Photocatalytic Mechanism

**Figure 6:**
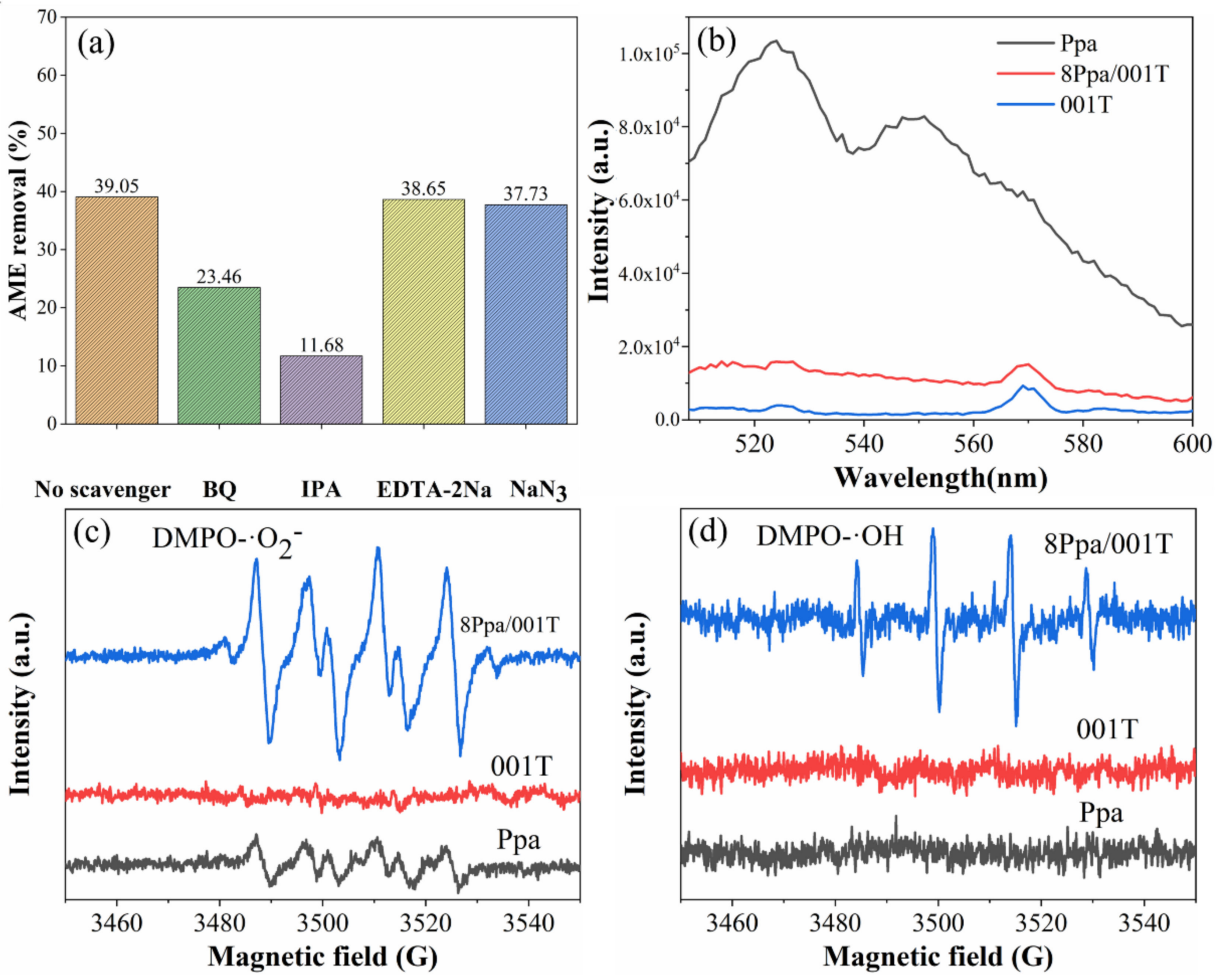
(**a**) Scavengers trapping experiments over the 8Ppa/001T sample; (**b**) Fluorescent probe results of the singlet oxygen; (**c**) The electron-spin resonance (ESR) tests for the detection of DMPO-•O_2_^−^; and (**d**) for the detection of DMPO-•OH under visible-light irradiation over the 001T, Ppa, and 8Ppa/001T samples.

To determine the degradation mechanism for AME over the optimized sample, several confirmatory tests were performed. As reported previously, the p-benzoquinone (BQ), Isopropanol (IPA), Ethylenediaminetetraacetic acid disodium salt (EDTA-2Na), and Sodium azide (NaN_3_) were widely used as scavengers for trapping the superoxide (•O_2_^−^), •OH, hole (h^+^), and singlet oxygen (^1^O_2_) species, respectively. Thus, we performed radical trapping experiments to confirm the dominant active species involved in the photodegradation of the AME pollutant. As seen in Figure 6a, in the absence of any scavenger, the removal rate is 39.05%. Worth noting, after the addition of various scavengers, the degradation is strongly inhibited in the presence of BQ and IPA. Meanwhile, the addition of EDTA-2Na and NaN_3_ scavengers have almost no effect on the degradation of AME. Interestingly, the degradation it strongly suppressed in the presence of IPA. Based on these investigations, it is demonstrated that both the •OH, and •O_2_^−^ radicals play a vital role in the degradation of the AME pollutant under visible-light irradiation.

Further, the fluorescent probe experiment for singlet oxygen (^1^O_2_) was measured using the fluorescent probe of SOSG, which effectively traps the ^1^O_2_ generated during photocatalysis [29]. The products generated after Singlet Oxygen Sensor Green (SOSG) react with ^1^O_2_ and gives a strong fluorescence response at the wavelength 520 nm. As seen in Figure 6b, the bare Ppa exhibits a weak signal of ^1^O_2_ after the photocatalytic reaction, due to the chlorophyll characteristics of Ppa itself. However, after loading 001T with Ppa, the 8Ppa/001T photocatalyst does not exhibit any signal corresponding to the ^1^O_2_, indicating that after coupling 001T with Ppa, the photogenerated electrons of Ppa are transferred to the conduction band of 001T and generated •O_2_^−^ radicals, which have strong oxidation ability.

To further elucidate the mechanism, the electron-spin resonance (ESR) technique was employed to detect the amount of •OH and •O_2_^−^ formed during the degradation of AME over the Ppa, 001T, and 8Ppa/001T samples under visible-light irradiation at room temperature. During the analysis, 5,5-dimethyl-1-pyrroline-*N*-oxide (DMPO) as a trapping reagent was added to the solution containing the photocatalyst and kept under stirring. As revealed in Figure 6c,d, no DMPO-•OH and DMPO-•O_2_^−^ adducts peak can be observed for the 001T sample, which is due to the fact that 001T cannot be excited under visible light. In addition, the bare Ppa reveals weak peaks of the corresponding DMPO-•O_2_^−^, but lack peaks of the DMPO-•OH. This is because of its proper conduction band and inappropriate valence band potentials for the generation of active radicals. In addition, the 8Ppa/001T sample reveals strong ESR DMPO-•O_2_^−^ and DMPO-•OH peaks, which further confirms the involvement of both these active intermediates in the degradation of the AME pollutant, demonstrating that the •OH signal produced mainly by 8ppa/001T is based on the reaction of •O_2_^−^ with water.

**Figure 7:**
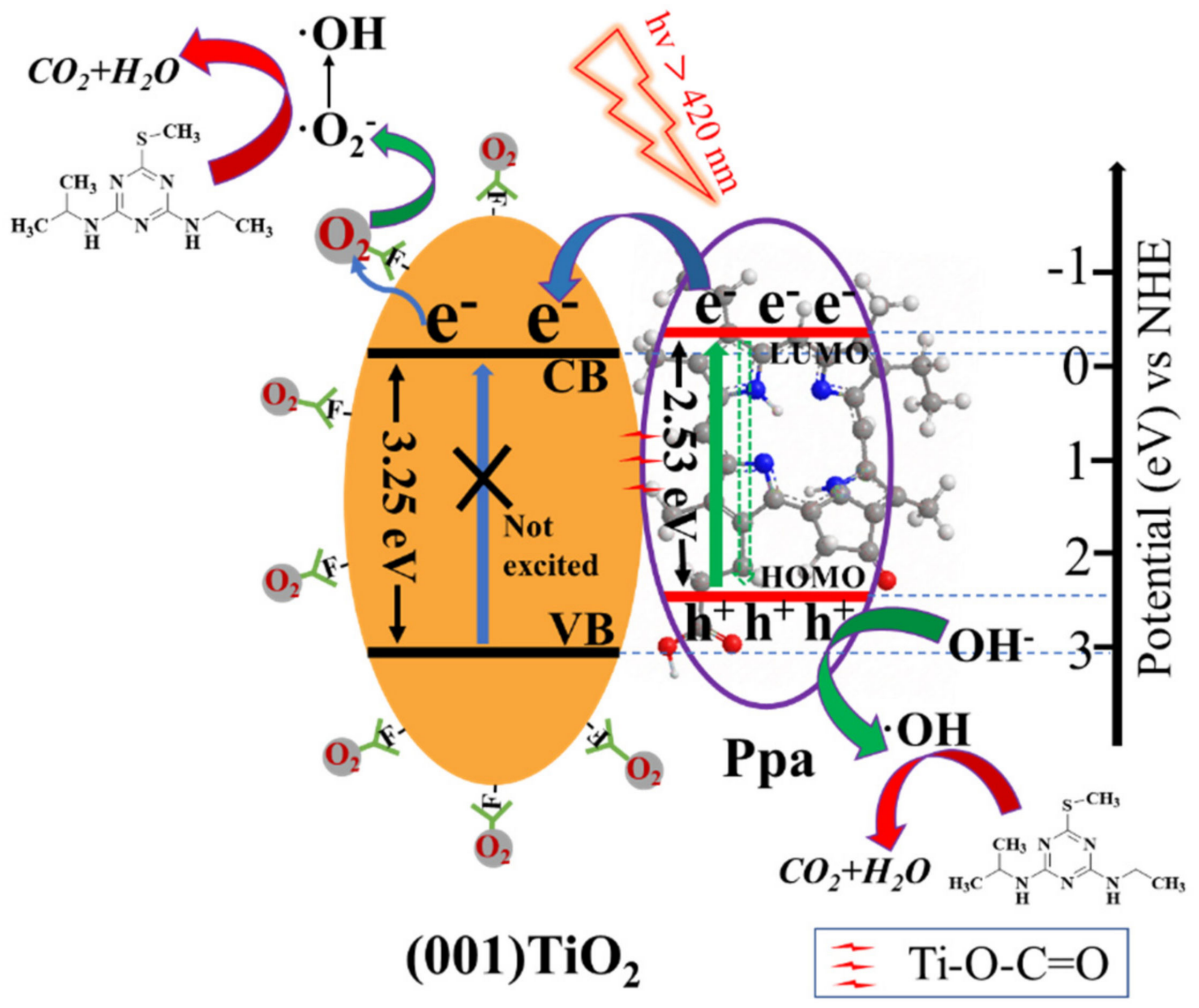
The schematic illustration of the Ppa/001T composite showing the charge carrier’s transfer and separation and the surface reactions.

To appraise the stability of the 8Ppa/001T photocatalyst, the photocatalytic recyclable test was performed under visible-light irradiation. For this, five photocatalytic cycles (each of 4 h) were completed. As seen in Figure S5, the degradation is not declined significantly even after five recycles, indicating that the resulting photocatalyst is highly stable during degradation. Based on the above results, we designed a scheme for charge transfer and separation and the photodegradation of AME over the 8Ppa/001T photocatalyst as revealed in Figure 7. The Mott–Schottky test (M-S) was conducted to estimate the flat band potential (E_fb_) of single 001T and Ppa at a frequency of 1 kHz. As shown in Figure S6a,b, the E_fb_ of 001T and Ppa are estimated to be −0.13 and −0.29 eV (vs. Ag/AgCl), equivalent to the 0.03 and −0.09 eV (vs. NHE). Moreover, the positive values of the slopes of M-S plots designate that both the 001T and Ppa are n-type semiconductors. In fact, the conduction band potential (E_CB_) value of the n-type semiconductor is ~0.2 eV negative than the E_fb_ [30], thus the E_CB_ of 001T and Ppa can be discerned to −0.13 and −0.29 eV (vs. NHE), respectively. According to the equation of E_VB_ = E_CB_ + E_g_, the valence band (E_VB_) potential of 001T and Ppa can be estimated to be 3.12 and 2.24 eV (vs. NHE), respectively, according to DRS and M-S results. As assured, the 001T cannot be excited under visible-light illumination because of its large band gap. Nevertheless, due to its negative conduction band potential, it can act as a suitable platform for accepting the visible-light excited electrons of Ppa in order to the drive the charge carrier’s separation and maintain the thermodynamic energy. It is demonstrated that the electrons in HOMO of Ppa are excited to LUMO under light irradiation and would relax faster to the bottom of LUMO and recombine with holes in the HOMO. After coupling with 001T, the partially excited electrons on LUMO of Ppa could thermodynamically transfer to the CB of 001T through the carbonyl bond group, leading to the enhanced charge separation. The electrons in the CB of 001T (−0.13 eV vs. NHE) could reduce oxygen to generate •O_2_^−^ (−0.046 eV vs. NHE) [31], and holes in the HOMO of Ppa (2.24 eV vs. NHE) could react with OH^-^ to produce •OH (1.99 eV vs. NHE) [32]. The oxygen adsorption improved by the F residues on the surface of the 001T catalyst effectively traps electrons and promotes charge separation while generating •O_2_^−^ radicals as the active species for the degradation of AME. During the degradation process, •O_2_^−^ and •OH radicals act as ROS and synergistically degrade AME.

### 2.5. Degradation Pathways

**Scheme 2:**
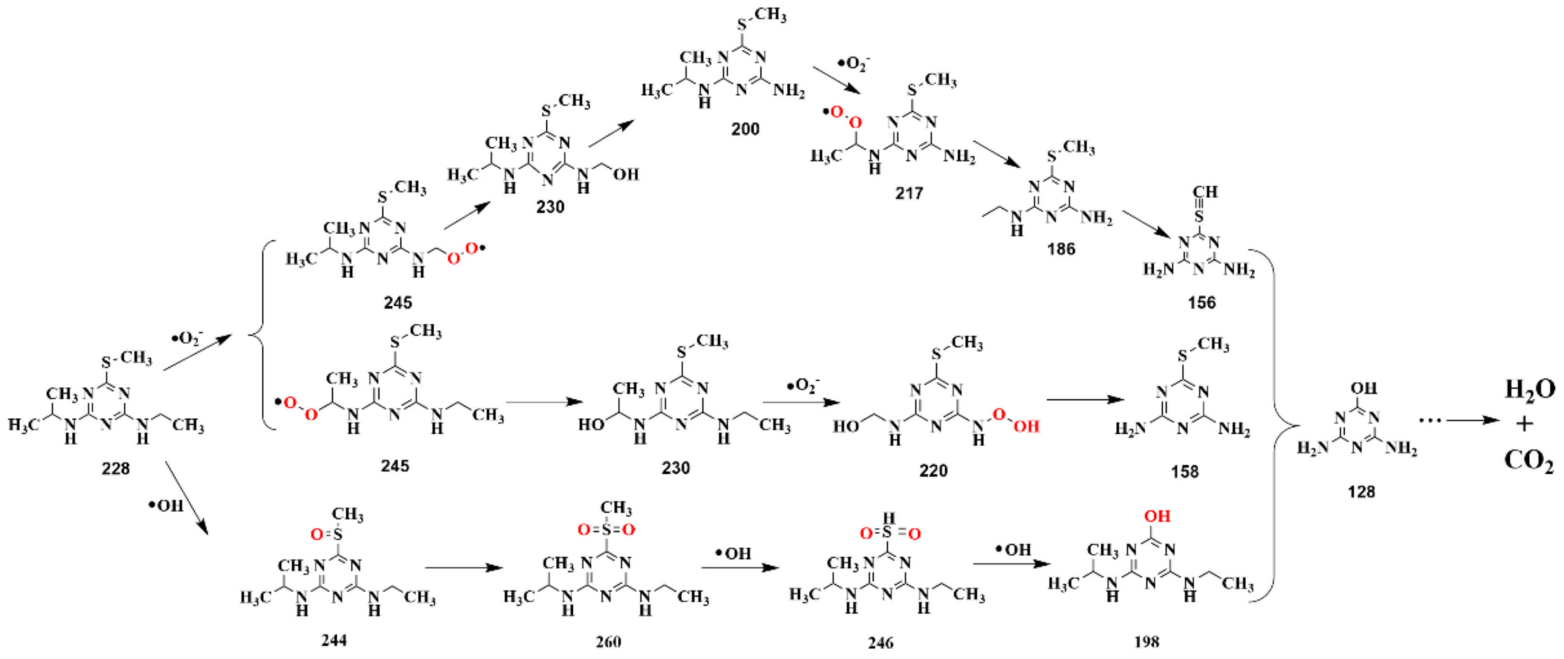
Proposed pathways for the photocatalytic degradation of AME by the dominant reactive intermediates produced over the 8Ppa/001T photocatalyst.

Based on the analysis of the above results, 8Ppa/001T exhibited good activity for photocatalytic degradation of AME, due to the involved •O_2_^−^ and •OH as the main active species. The synergistic effect of these two free radicals leads to an increase in the photocatalytic performance of 8Ppa/001T for degradation of the AME pollutant. However, the AME degradation pathway via the •O_2_^−^ and •OH radicals is unclear and seldom investigated. To clarify this, we used the LC-MS/MS technique to identify the intermediates formed during the photocatalytic degradation of AME and verified by further analysis of the second-order mass spectrum (Figure S7A–R). Consequently, both the •O_2_^−^ and •OH were the predominant radicals involved in the AME degradation. Thus, by analyzing the fragments via the LC-MS/MS technique, two different pathways are proposed. Initially, the •O_2_^−^ radical attacks the triazine ring as depicted in Scheme 2. Since the AME is composed of two alkyl chains, the •O_2_^−^ radical will attack the two alkyl chains in succession, but the order is not certain. Three important fragments with *m*/*z* values of 245, 230, and 200 were identified (Figure S7B–D), which are the distinctive intermediates formed as a result of the attack of •O_2_^−^ radicals. Thus, one of the methyl groups is transformed to the hydroxyl group. Along with the continuous attack of •O_2_^−^ radicals, the alkyl chains at the other end are also oxidized to form hydroxyl groups, as confirmed by the detected intermediates with *m*/*z* values of 217, 186, and 156 (Figure S7E–G). Worth noting, when the •O_2_^−^ radicals attacks the side of the isopropyl chain, they could also produce fragments with *m*/*z* values of 245 and 230 (Figure S7B,C), but structurally isomerized as before. The continuous attack of •O_2_^−^ radicals leads to the hydroxylation on one side of the ethyl alkyl chain, based on the detected intermediates with *m*/*z* values of 220 and 158 (Figure S7H,I). Another reaction pathway involves the attack of •OH radicals. As revealed in Scheme 2, the •OH radicals first attack the -S-CH_3_ group of the triazine ring, and generate a fragment with *m*/*z* value of 244 (Figure S7J). In the next steps, •OH radicals react with the -S-CH_3_ group until finally replaced by the -OH, as confirmed from the detected intermediates with *m*/*z* values of 260, 246, and 198 (Figure S7K–M). It is important to note that the final intermediate product formed via the reaction of both •O_2_^−^ and •OH radical pathways is Ammeline with *m*/*z* value of 128 (Figure S7N). Lastly, the opening of the ring takes place and small fragments with *m*/*z* values of 96, 102, 86, and 74 (Figure S7O–R) are produced. Based on the above analysis, it is concluded that the •O_2_^−^ and •OH radicals are the dominant active species involved in the photocatalytic degradation of AME over the 8Ppa/001T photocatalyst and both of the radicals are crucial for transformation of the AME into open ring small fragments.

### 2.6. Mechanism Discussion

In order to further speculate on the intermediate products of the two degradation routes, the experiments for change in the intensity of each intermediate product fragments with degradation time were measured as shown in Figure S8. As revealed in Figure S8a–c, the intensity of all intermediate product fragments in the degradation route of •O_2_^−^ and •OH radicals have remarkably decreased or even disappeared after 240 min, indicating that AME is nearly mineralized after 240 min. The trend of the fragmentation changes illustrates the speed of the two free radical attack routes. As seen in Figure S8a,b, the first fragment of *m*/*z* = 245 representing the route of the •O_2_^−^ radicals attack reached the highest value at 120 min, and other fragments reached the highest value at 150 min, and then showed a downward trend. Meanwhile, as showed in Figure S8c, the first fragment of *m*/*z* = 244 representing the route of the •OH radicals attack reached a highest value at 150 min, and other fragments showed a downward trend after 180 min, indicating that the degradation route of •O_2_^−^ radicals started first and reacted faster than the •OH radicals. In the •O_2_^−^ and •OH radicals degradation routes, •O_2_^−^ radicals first attack AME, triggering the occurrence of degradation reactions, and the •OH radicals achieve better mineralization of AME because of its higher oxidation capacity. This is consistent with the free radical formation mechanism. In Figure S8d, the intensity change trend of all ion fragments after the ring opening can be seen confirming its low content after 180 min.

Figure S9a,b reveals the change trend of the ion concentration after the addition of isopropanol and BQ scavengers, respectively. As seen in Figure S9a,b, after the addition of IPA and BQ, the change trend of the fragments is different from the previous as mentioned in Figure S8. In the data of the trend of intermediate products degraded with •OH as the reactive group, the inhibition by the addition of isopropanol caused the fragments with *m*/*z* = 244 and 260 to show a trend of increasing intensity all the time, while the fragments with *m*/*z* = 246 and 198 were in a slow decreasing trend and still had a high intensity after 240 min. Similarly, in the data of the trend of intermediate products degraded with •O_2_^−^ as the reactive group, the inhibition by the addition of BQ led to a trend of increasing intensity all the time for the fragments with *m*/*z* = 245, 156, and 220, while the fragments with *m*/*z* = 230, 200, 186, and 158 were in a slow decreasing trend and still had a high intensity after 240 min. Thus, based on the above discussion, it is demonstrated that the performance of the 8Ppa/001T photocatalyst for AME degradation is accredited to the remarkably promoted charge separation and synergistic degradation with •OH and •O_2_^−^.

## 3. Materials and Methods

### 3.1. Materials

Tetrabutyl titanate (Ti(Obu)_4_), hydrofluoric-acid (HF, 40%), anhydrous ethanol (EtOH), EDTA-2Na, IPA, BQ, and NaN_3_ were obtained from Sinopharm Chem. (Shanghai, China). Reagent Co., Ltd. (Beijing, China). Ppa, DMPO, Coumarin, and SOSG were obtained from Aladdin Industry Inc. (Nashville, TN, USA). Note: all the chemical reagents were of high purity analytical grade. The de-ionized water was used as a solvent in all experiments.

### 3.2. Preparation of 001T

The anatase 001T was prepared via a fluorine induced hydrothermal method [33]. Then, 20 mL EtOH was taken and 5 mL of Ti(Obu)_4_ was added to it and then vigorously stirred. After that, 0.9 mL of HF was dropwise added to it and the solution was vigorously stirred for 1 h. Finally, it was transferred into a 25 mL volume stainless-steel Teflon lined autoclave, and treated at 160 °C for 24 h. After room temperature was attained, the precipitate (white) was centrifugated and washed with ethanol and deionized water for several times, then dried at 60 °C for 12 h, and named as 001T.

### 3.3. Synthesis of xPpa/001T

The 001T nanosheets were modified by using Ppa via the incipient wetness impregnation method. A total of 30 mL ethanol was taken and 0.5 g of 001T was dispersed in it. Then, different percentage ratios by mass (i.e., 2, 4, 6, 8, and 10%) of Ppa were added to the above dispersion and stirred for 24 h in the dark at room temperature. The composite samples were collected by centrifugation at 8000 rpm for 10 min. The obtained catalysts were labeled as xPpa/001T, where x is weight percentages of Ppa.

### 3.4. Characterizations

A Bruker D-MAX-2500 (Rigaku, Tokyo, Japan) X-ray diffractometer (XRD) was used for X-ray diffraction analysis of the samples. A Shimadzu UV-2770 UV-vis spectrophotometer was used for diffuse reflectance spectra analysis. A Bruker Equinox-55 Fourier-transform infrared spectrometer was used for FT-IR spectra analysis. The Kratos-AXIS ULTRA-DLD X-ray photoelectron spectrometer was used for X-ray photoelectron spectra analysis. A JEOL-JEM-2010 instrument was used for morphology analysis of the samples. The FEI-Tecnai (G2 S-Tw) equipped with energy-dispersive X-ray (EDX) sensor was used for elemental analysis. A home-built instrument connected to a lock-in amplifier (SR-830) coordinated to a light chopper (SR-540) was used for surface photovoltage spectra (SPS) analysis. A FLS-920 (Edinburgh) instrument was used for measuring the time-resolved photoluminescence (TR-PL) spectra. A model Bruker-EMX-plus spectrometer was used for conducting the electron spin resonance (ESR) tests. Other experimental details are provided in the supporting information.

### 3.5. Evaluation of •OH

For •OH measurement, 0.05 g photocatalyst was dispersed in 50 mL coumarin aqueous solution (0.001 mol L^−1^). The mixture was stirred in dark condition for 10 min to achieve adsorption–desorption equilibrium and then irradiated under a 150 W (GYZ220) high-pressure Xe-lamp under visible-light (λ > 400 nm) for 1 h. Each mixture was centrifuged and the supernatant was taken in a cuvette for detecting the 7-hydroxycoumarin at excitation wavelength of 332 nm and emission of 460 nm through a Perkin-Elmer LS-55 spectrofluorometer.

### 3.6. Photocatalytic Degradation Experiments

For photocatalytic degradation of AME, a 500 W xenon arc lamp (λ > 400 nm, Solar-500, Beijing Perfect-light., Ltd., Beijing, China) with the average light-intensity of 100 mW cm^−2^ was used as an irradiation source. In photocatalytic experiments, 0.2 g of each catalyst and AME solution (i.e., 50 mL from the stock 10 mg L^−1^) was added to a 250 mL beaker and stirred in the dark for 0.5 h to attain adsorption–desorption equilibrium. Then, each solution was illuminated under visible light and 1 mL of the solution was taken out at specific interval, filtered through a 0.22 μm nylon membrane and its concentration was analyzed. To confirm the reproducibility, the experiments were repeated thrice and the mean values were selected. For stability confirmation, the recyclable tests of the photocatalysts were carried out.

### 3.7. Analytical Methods

The monitoring of the decrease in concentration of AME was analyzed via a Shimadzu-20A high-performance liquid-chromatography (HPLC) using a C18 column (250 × 4.6 mm 5.0 μm from Agilent). The mobile phase consisted of 30:70 (*v*/*v*) mixture of water and methanol, injected with a flow-rate of 1.0 mL min^−1^ and analyzed at UV absorbance wavelength of 225 nm. In these parameters, the peak of AME appeared at 8.27 min, showing a limit of detection (LOD) = 0.020 mg L^−1^.

To identify the photodegradation intermediates of the AME, samples were analyzed by using a HPLC system (Waters Series H-Class, Waters Technologies, Milford, MA, USA) coupled to LC-MS/MS (TQD, Waters Technologies) tandem mass spectrometry with electrospray-ionization (ESI) interface operated at a positive-ionization mode. The conditions used in the spectrometry were as follows: capillary 3.5 KV; cone 30 V; desolvation temperature of 350 °C; source gas flow 800 L/Hr; collision energy MS 10–20 eV; Mass spectrum scanning range 30–500 *m*/*z*. The intermediates fragments were investigated via the full scan mode using positive ESI source. The second-order mass spectrum fragments were detected via the product ion scan mode. The HPLC conditions were the same as previously used to monitor degradation of AME.

### 3.8. Scavenging Trapping Experiments

To confirm the role of •OH, •O_2_^−^, h^+^, and ^1^O_2_ in the photocatalytic degradation mechanism, IPA, (10 mmol L^−1^), BQ, (6 mmol L^−1^), EDTA-2Na (1 mmol L^−1^), and NaN_3_ (75 mmol L^−1^) were added to the AME solution (50 mL, 10 mg L^−1^), respectively. The scavenger’s concentration was based on previous research [34,35].

## 4. Conclusions

In summary, 001T modified Ppa composite was successfully fabricated via the self-assembly approach of OH induced. The photoactivity of the photocatalysts was evaluated by the degradation of AME pollutant under visible-light irradiation. The optimized 8Ppa/001T sample revealed 41-fold enhanced activity for AME degradation compared to the bare Ppa sample. The enhanced performance of the composite is accredited to the improved charge separation by coupling 001T, which acts as a proper thermodynamic platform for accepting the highly energetic electrons of Ppa and to the improved surface O_2_ adsorption via the residual fluoride. In addition, the simultaneous involvement of •O_2_^−^ and •OH radicals in the degradation of AME was confirmed via the analysis of various techniques. This work is of utmost importance since it gives us detailed information regarding the charge carrier’s separation and the impact of the radical pathway that will pave a new approach toward the design of high activity visible-light driven photocatalysts.

## Data Availability

Not applicable.

## Supplementary Materials

The following supporting information can be downloaded at: https://www.mdpi.com/article/10.3390/molecules27175576/s1, Table S1: Volume and absorbance of supernatant for different catalysts, and calculated mass of Ppa in supernatant and loading of Ppa. Table S2: The fitted parameters of the achieved time-resolved PL spectra. Figure S1: XRD patterns of 001T, Ppa, and xPpa/001T. Figure S2: (a) Full XPS spectra of 8Ppa/001T, 001T, and Ppa; (b) O1s fine spectra of XPS of 8Ppa/001T and 001T; (c) C1s spectra of XPS of Ppa; (d) N1s spectra of XPS of Ppa. Figure S3: Photoelectrochemical I-T curves of the 001T, Ppa, and 8Ppa/001T samples. Figure S4: (a) Plot of the ln(C/Co) versus time for photodegradation of AME; (b) the effect of inland lake water and local tap water (used for preparation of AME solution, 10 mg L^−1^) on the degradation rate of AME over the 8Ppa/001T catalyst (content = 4 g L^−1^) at pH 6. Figure S5: Photocatalytic recyclable test for AME degradation over the 8Ppa/001T sample under visible-light irradiation. Figure S6: Mott–Schottky curves of (a) 001T and (b) Ppa. Figure S7A–R: Extract ion chromatography (EIC) analysis of the reaction intermediates for degradation of AME over 8Ppa/001T photocatalyst and the product ion scan spectrometry of identified reaction intermediates (inset). Figure S8: Fragment change trend chart. Figure S9: (a) Trends of individual ionic fragments after the addition of isopropanol scavengers; (b) Trends of individual ionic fragments after the addition of benzoquinone scavengers.

## Sample Availability

Samples of the compounds are available from the authors.
