# Supplementary material for: Pyropheophorbide-a/(001) TiO2 Nanocomposites with Enhanced Charge Separation and O2 Adsorption for High-Efficiency Visible-Light Degradation of Ametryn
Source: Molecules. 2022 Aug 30;27(17):5576. doi: 10.3390/molecules27175576 (PMC9458040; doi:10.3390/molecules27175576)
Supplement: Supplementary file 1 [file molecules-27-05576-s001.zip › molecules-1855476-supplementary.pdf]

# Supporting Information

# Pyropheophorbide-a/(001) $TiO_2$ Nanocomposites with Enhanced Charge Separation and $O_2$ Adsorption for High-Efficiency Visible-Light Degradation of Ametryn

Songtao Liu [1,†], Rui Yan [1,*,†], Muhammad Humayun [2], Huanli Zhang [1],

Yang Qu [3,*] and Yingxue Jin [1,4,*]

1 Key Laboratory for Photonic and Electronic Bandgap Materials, Ministry of Education, College of Chemis-try & Chemical Engineering, Harbin Normal University, Harbin 150025, China

2 Wuhan National Laboratory for Optoelectronics, Optics Valley Laboratory, School of Optical & Electronics Information, Huazhong University of Science & Technology, Wuhan 430074, China

3 Key Laboratory of Functional Inorganic Materials Chemistry (Ministry of Education), School of Chemistry and Materials Science, International Joint Research Center and Lab for Catalytic Technology, Heilongjiang University, Harbin 150080, China

4 Key Laboratory of Molecular Cytogenetics and Genetic Breeding of Heilongjiang Province, College of Life Science and Technology, Harbin Normal University, Harbin 150025, China

* Correspondence: yanrui-1981@163.com (R.Y.), quyang@hlju.edu.cn (Y.Q.); jyxprof@163.com (Y.J.)

† These authors contributed equally to the work.

**Experimental**

*1. Single line state oxygen test*

Singlet Oxygen Sensor Green (SOSG) was used as a specific probe for the production of singlet oxygen in the photocatalytic degradation of atrazine by fluorescence spectroscopy. The catalyst was dispersed in 6 mL of anhydrous ethanol with 0.001 g of catalyst, 10 μL of prepared SOSG in methanol and shaken well.

*2. Electron spin resonance (ESR) Test*

Electron spin resonance (ESR) was used to determine whether reactive species such as •OH and $•O_2^-$ were produced during the reaction. Measurements of reactive species produced during the reaction were recorded using 5,5-dimethyl-1-pyrroline-N-oxide (DMPO) as a trapping agent. The •OH and $•O_2^-$ were tested in water and methanol systems, respectively. Tests were obtained by Bruker A300-10/12 (model and origin) under visible light at room temperature.

*3. Photoelectrochemical test*

The photocurrent tests (I-T), Mott-Schottky curves (M-S) and electrochemical impedance spectra (EIS) of the obtained products were investigated in a conventional three-electrode model on an electrochemical workstation (CHI760D, Shang Hai, China). A Pt coil was employed as the counter electrode, Ag/AgCl electrode served as the reference electrode and 0.5 M $Na_2SO_4$ solution (pH = 7) was used as the electrolyte under room temperature, while the FTO with photocatalysts coated on as the working electrode. The working electrodes were prepared by electrophoretic

deposition in acetone solution containing 12 mg of the sample powder and 3 mg of iodine. The slurry for the working electrode (1 cm x1 cm) prepared by electrophoretic deposition in acetone solution containing 12 mg of samples and 3 mg of iodine. After that, the working electrode annealed at 300 °C for 20 min. The photocurrents were measured using 0.5V bias voltage under light illumination of the 300W Xe lamp. EIS curves were recorded under the open circuit voltage in the frequency range of 102–106 Hz with an ac amplitude of 10 mV.

*4. Precise loading amount of Ppa*

The amount of Ppa in the supernatant solution can be calculated by Eq. (1) as follows:

$$X_{Ppa}=(A_{667}-0.0088)/44.996\ (mg\ mL^{-1}) \quad (1)$$

Where, $A_{667}$ is absorbance at 667 nm measured by using UV-vis spectrophotometer in anhydrous ethanol. $X_{Ppa}$ is the amount of Ppa in the supernatant solution.

The precise loading amount of Ppa can be calculated by Eq. (2) as follows:

$$Y_{Ppa}=B_{Ppa}-X_{Ppa} \quad (2)$$

where $B_{Ppa}$ is the amount of Ppa added during the synthesis of xPpa/001T. $Y_{Ppa}$ is the precise loading amount of Ppa. Based on the UV-Vis absorption spectra results, the precise loading amount of the Ppa on $TiO_2$ calculated as being 0.8%, 1.5%, 2.6%, 3.2%, 3.4%. Detailed data is recorded in Table S1.

**Table S1.** Volume and absorbance of supernatant for different catalysts, and calculated mass of Ppa in supernatant and loading of Ppa.

| Catalysts | Volume of supernatant(mL) | Absorbance of supernatant | The quality of Ppa in the supernatant (mg) | The loaded amounts of Ppa (%) |
|---|---|---|---|---|
| 2Ppa/001T | 220 | 1.292 | 6.272 | 0.740 |
| 4Ppa/001T | 240 | 2.345. | 7.539 | 1.485 |
| 6Ppa/001T | 220 | 3.609 | 17.603 | 2.419 |
| 8Ppa/001T | 236 | 4.626 | 24.217 | 3.06 |
| 10Ppa/001T | 252 | 5.629 | 31.476 | 3.572 |

**Table S2.** The fitted parameters of the achieved time-resolved PL spectra.

| Samples | $A_1$ | $\tau_1$ | $A_2$ | $\tau_2$ | $A_3$ | $\tau_3$ | $\tau$（ns） |
|---|---|---|---|---|---|---|---|
| Ppa | 0.4579 | 1.3121 | 0.4289 | 4.5738 | 0.0749 | 17.3432 | 8.363 |
| 8Ppa/001T | 0.9890 | 0.2314 | 0.3325 | 1.2723 | 0.0469 | 7.0756 | 5.161 |

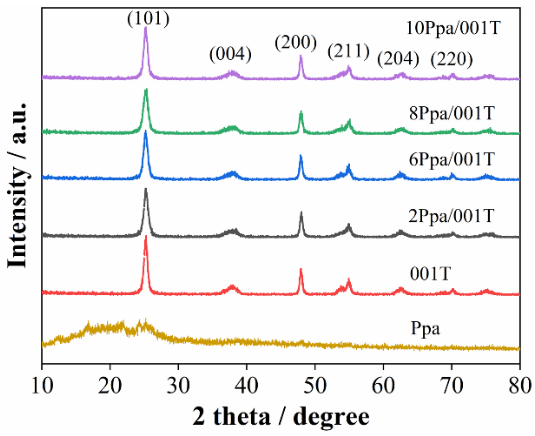


**Figure S1.** XRD patterns of 001T, Ppa and xPpa/001T.

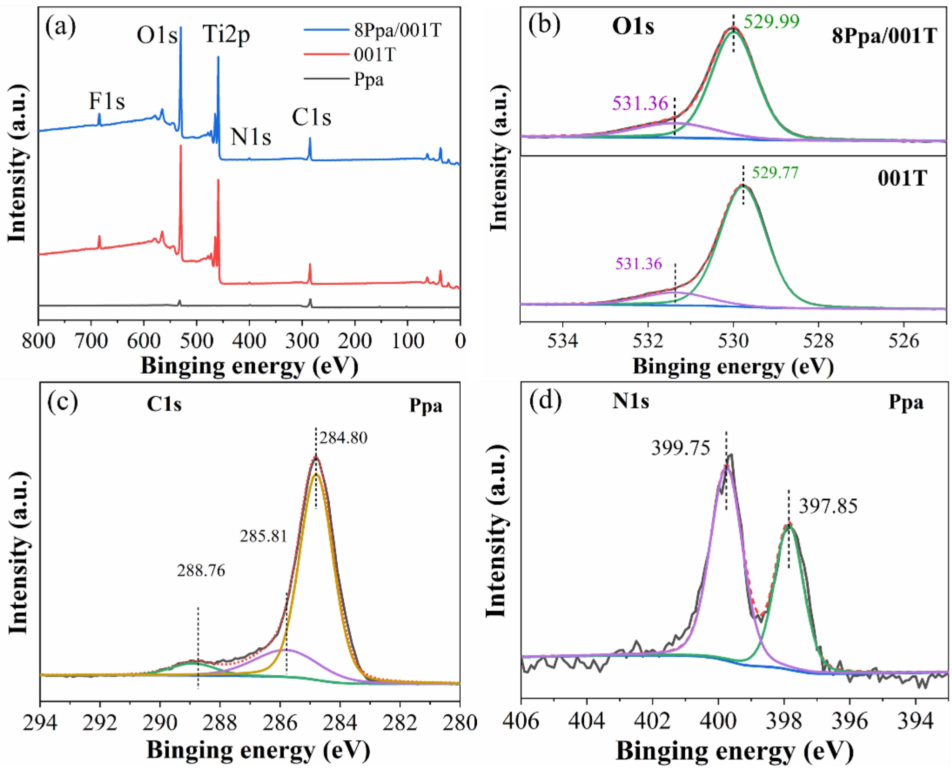


**Figure S2.** (a) Full XPS spectra of 8Ppa/001T, 001T and Ppa; (b) O1s fine spectra of XPS of 8Ppa/001T and 001T; (c) C1s spectra of XPS of Ppa; (d) N1s spectra of XPS of Ppa.

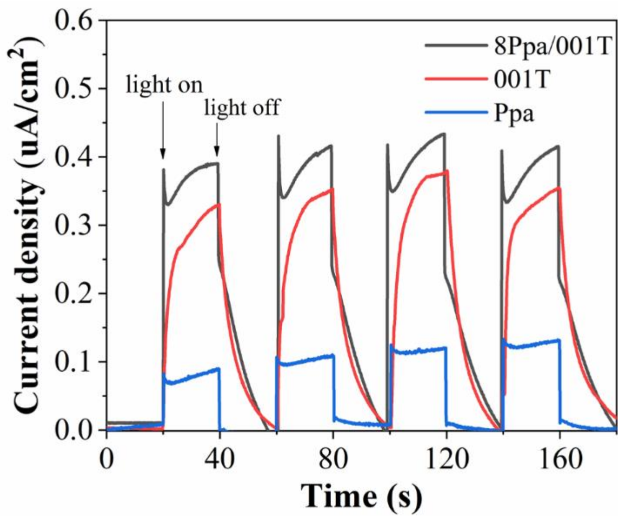


**Figure S3.** Photoelectrochemical I-T curves of the 001T, Ppa and 8Ppa/001T samples.

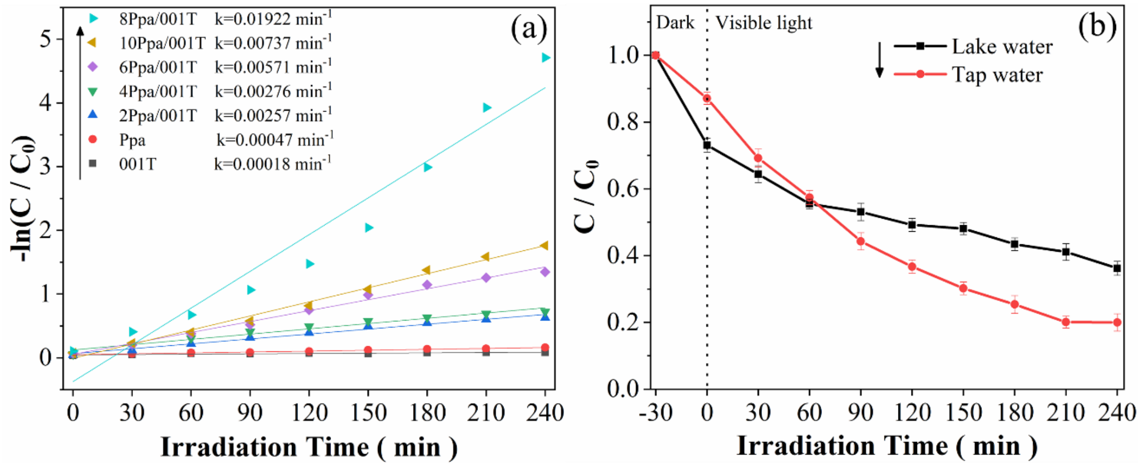


**Figure S4.** (a) Plot of the ln($C/C_o$) versus time for photodegradation of AME; (b) the effect of inland lake water and local Tap water (used for preparation of AME solution, 10 mg $L^{-1}$) on the degradation rate of AME over the 8Ppa/001T catalyst (content = 4 g $L^{-1}$) at pH 6.

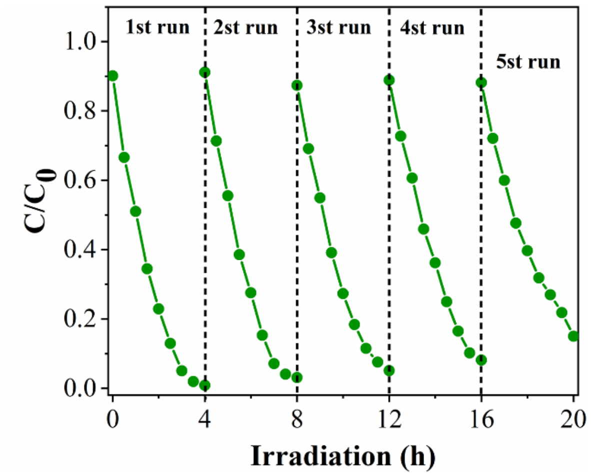


**Figure S5.** Photocatalytic recyclable test for Ametryn degradation over the 8Ppa/001T sample under visible light irradiation.

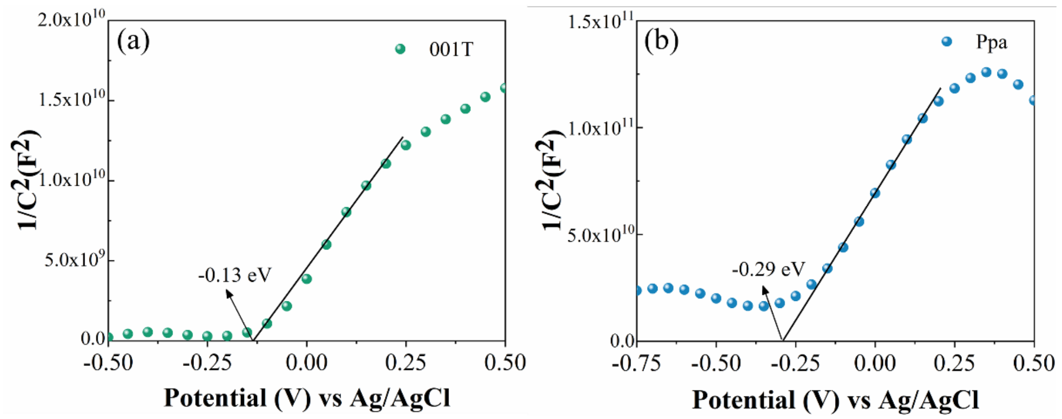


**Figure S6.** Mott-Schottky curves of (a) 001T and (b) Ppa.

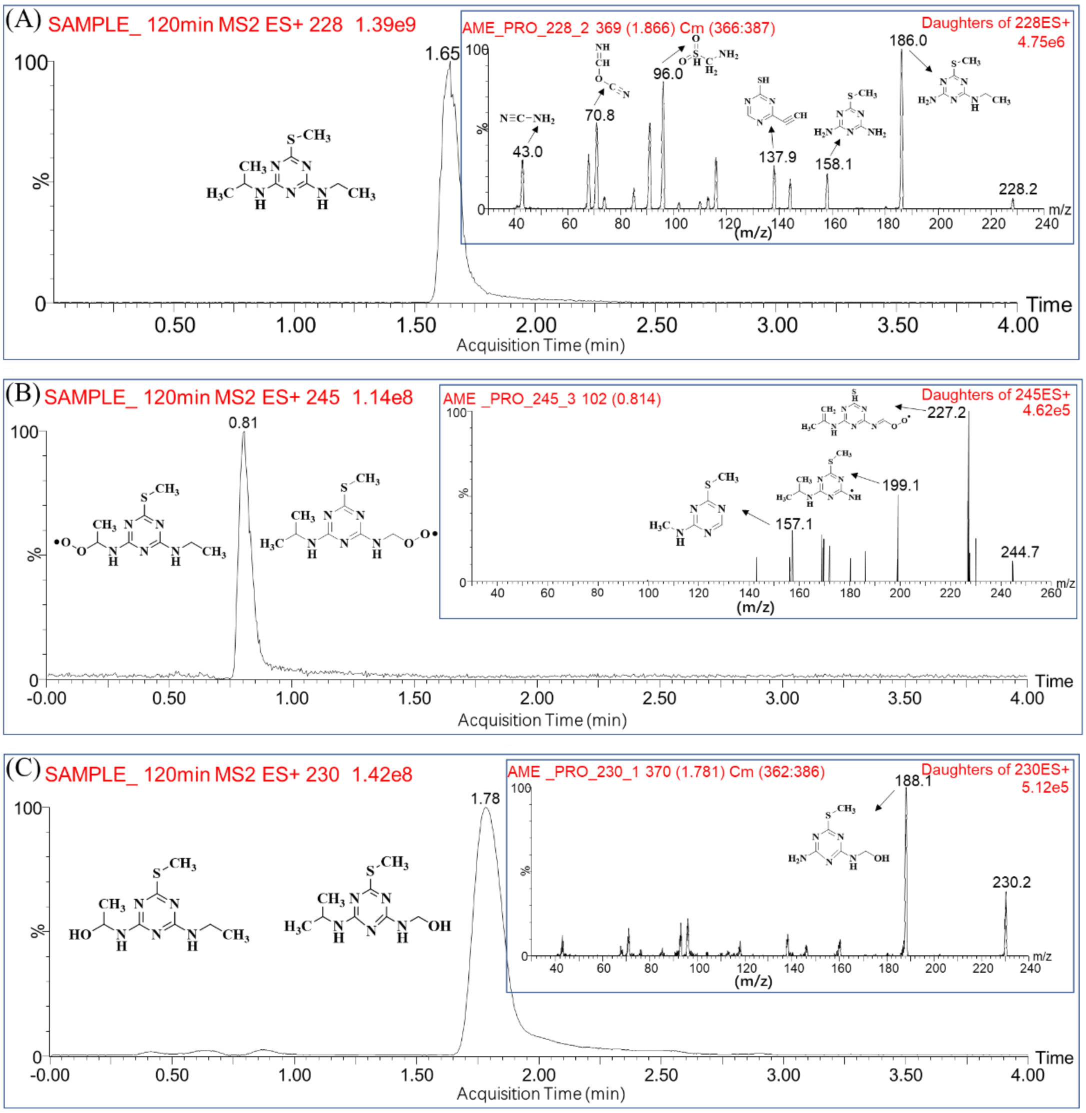

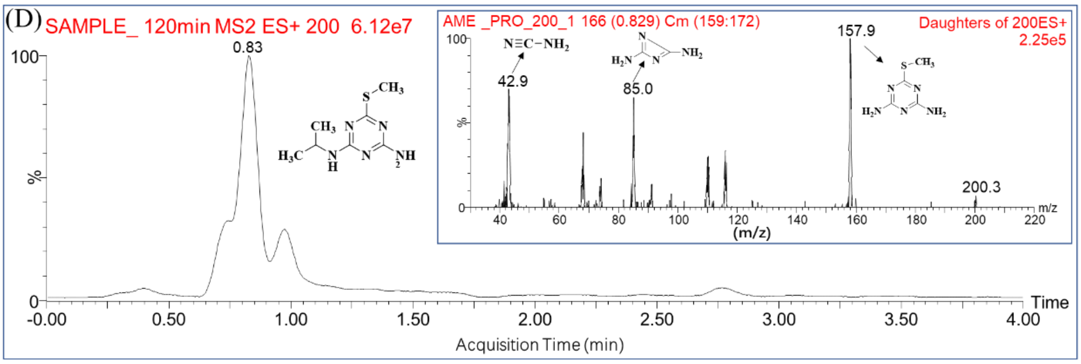
(D) SAMPLE_ 120min MS2 ES+ 200 6.12e7
0.83
AME _PRO_200_1 166 (0.829) Cm (159:172)
Daughters of 200ES+
2.25e5
42.9
85.0
157.9
200.3
(m/z)
Acquisition Time (min)

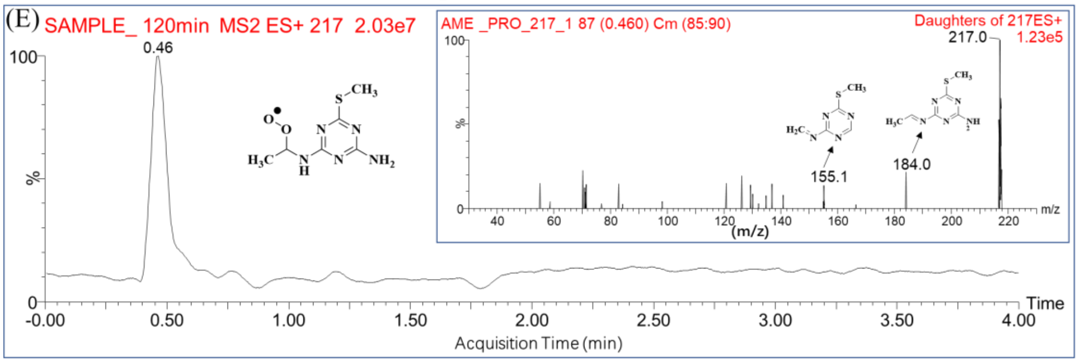
(E) SAMPLE_ 120min MS2 ES+ 217 2.03e7
0.46
AME _PRO_217_1 87 (0.460) Cm (85:90)
Daughters of 217ES+
1.23e5
217.0
155.1
184.0
(m/z)
Acquisition Time (min)

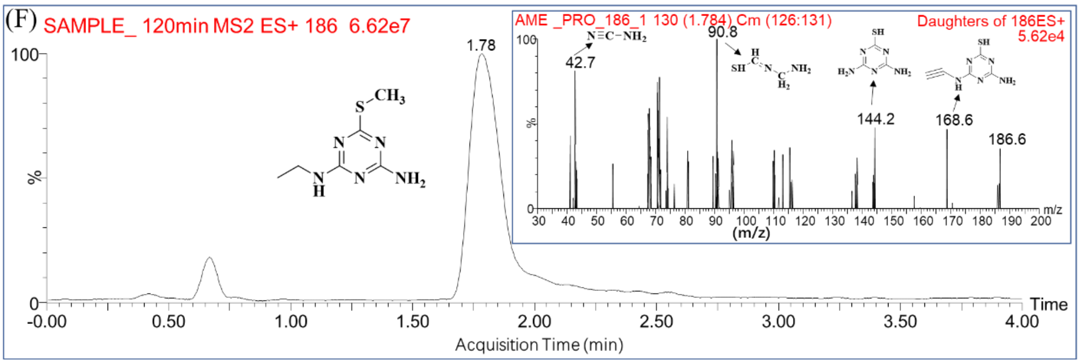
(F) SAMPLE_ 120min MS2 ES+ 186 6.62e7
1.78
AME _PRO_186_1 130 (1.784) Cm (126:131)
Daughters of 186ES+
5.62e4
42.7
90.8
144.2
168.6
186.6
(m/z)
Acquisition Time (min)

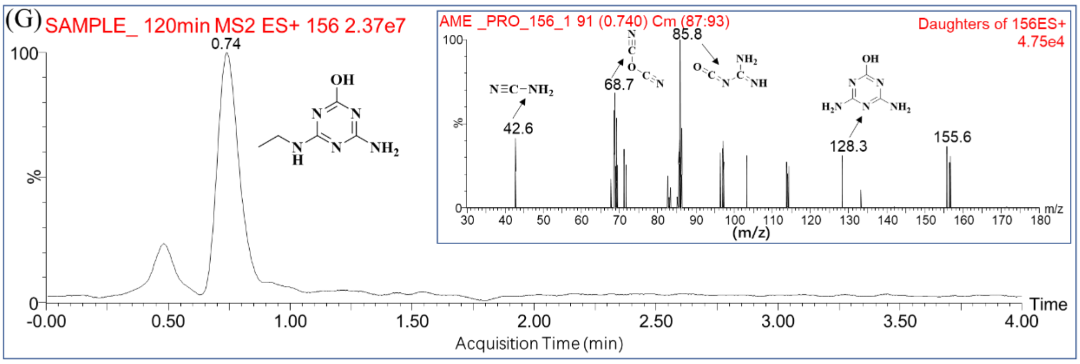
(G) SAMPLE_ 120min MS2 ES+ 156 2.37e7
0.74
AME _PRO_156_1 91 (0.740) Cm (87:93)
Daughters of 156ES+
4.75e4
42.6
68.7
85.8
128.3
155.6
(m/z)
Acquisition Time (min)

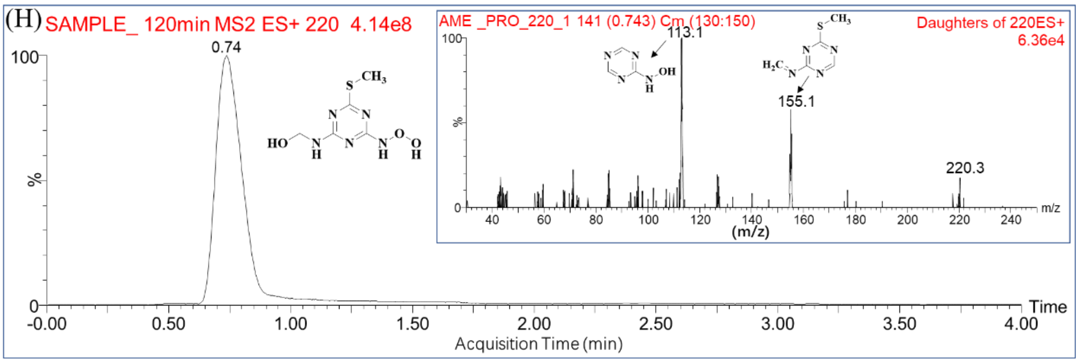
(H) SAMPLE_ 120min MS2 ES+ 220 4.14e8
0.74
AME _PRO_220_1 141 (0.743) Cm (130:150)
Daughters of 220ES+
6.36e4
113.1
155.1
220.3
(m/z)
Acquisition Time (min)

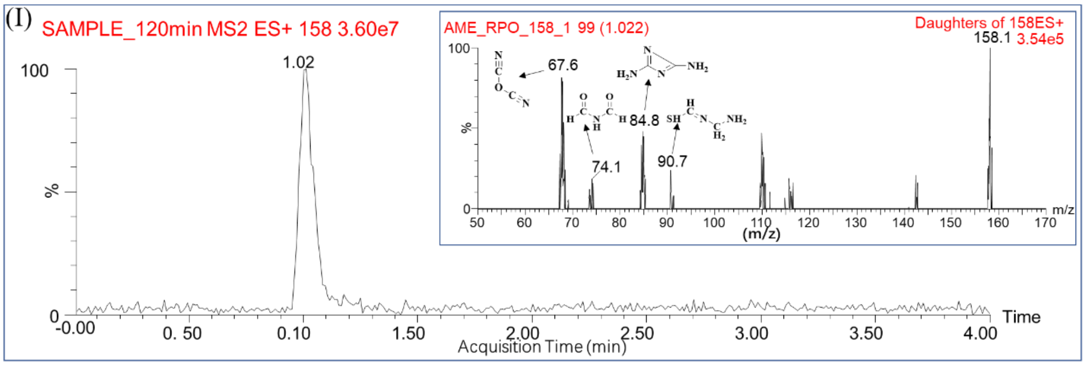
(I) SAMPLE_120min MS2 ES+ 158 3.60e7
1.02
AME_RPO_158_1 99 (1.022)
Daughters of 158ES+
158.1 3.54e5
67.6
74.1
84.8
90.7
(m/z)
Acquisition Time (min)

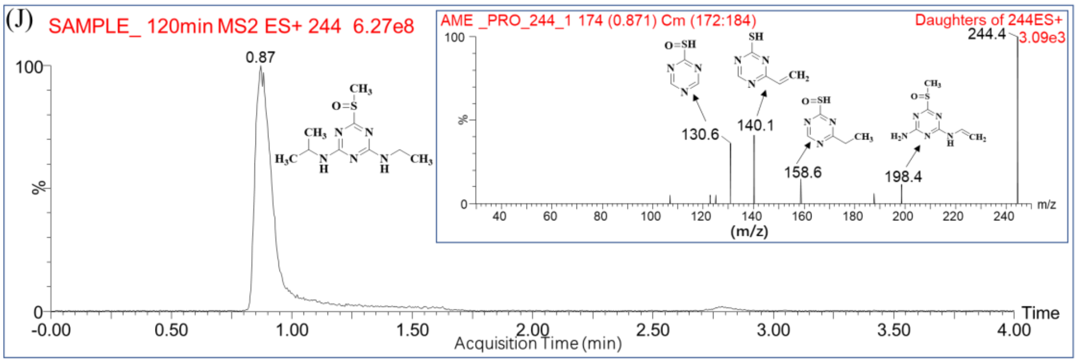
(J) SAMPLE_ 120min MS2 ES+ 244 6.27e8
0.87
AME _PRO_244_1 174 (0.871) Cm (172:184)
Daughters of 244ES+
244.4 3.09e3
130.6
140.1
158.6
198.4
(m/z)
Acquisition Time (min)

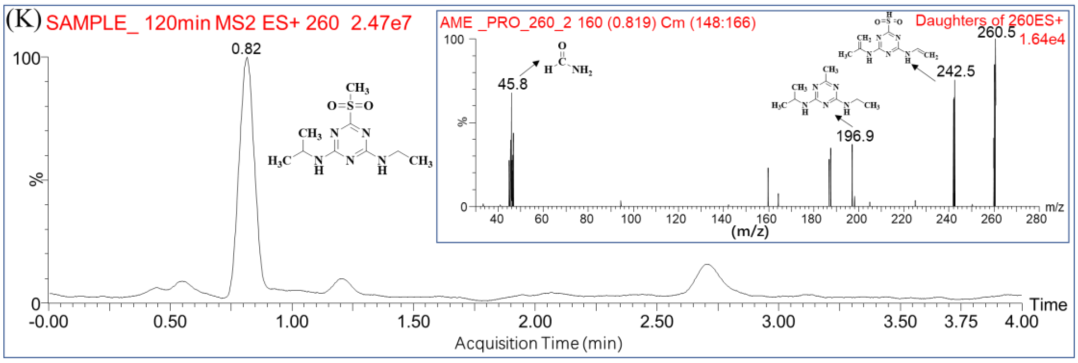
(K) SAMPLE_ 120min MS2 ES+ 260 2.47e7
0.82
AME _PRO_260_2 160 (0.819) Cm (148:166)
Daughters of 260ES+
260.5 1.64e4
45.8
242.5
196.9
(m/z)
Acquisition Time (min)

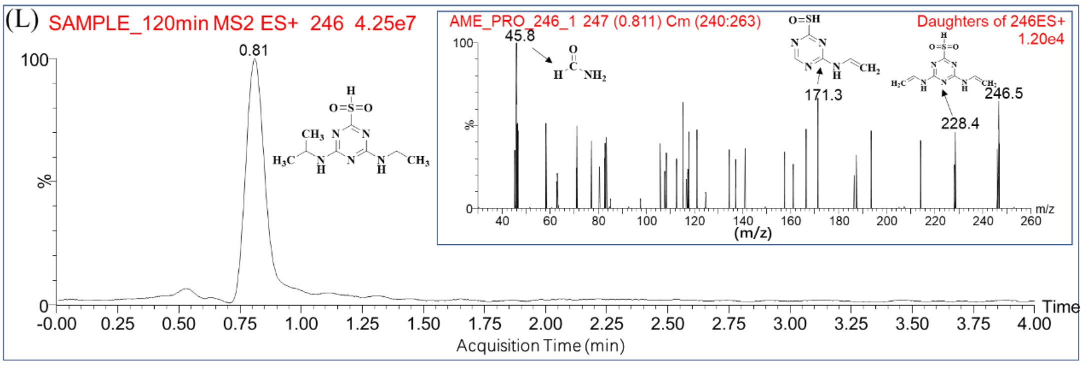
(L)
SAMPLE_120min MS2 ES+ 246 4.25e7
0.81
Acquisition Time (min)
Time
AME_PRO_246_1 247 (0.811) Cm (240:263)
Daughters of 246ES+
1.20e4
45.8
171.3
228.4
246.5
(m/z)

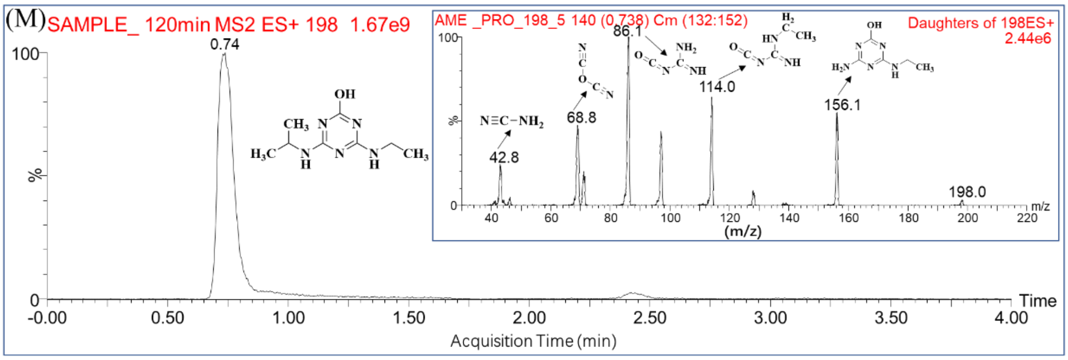
(M)
SAMPLE_ 120min MS2 ES+ 198 1.67e9
0.74
Acquisition Time (min)
Time
AME _PRO_198_5 140 (0.738) Cm (132:152)
Daughters of 198ES+
2.44e6
42.8
68.8
86.1
114.0
156.1
198.0
(m/z)

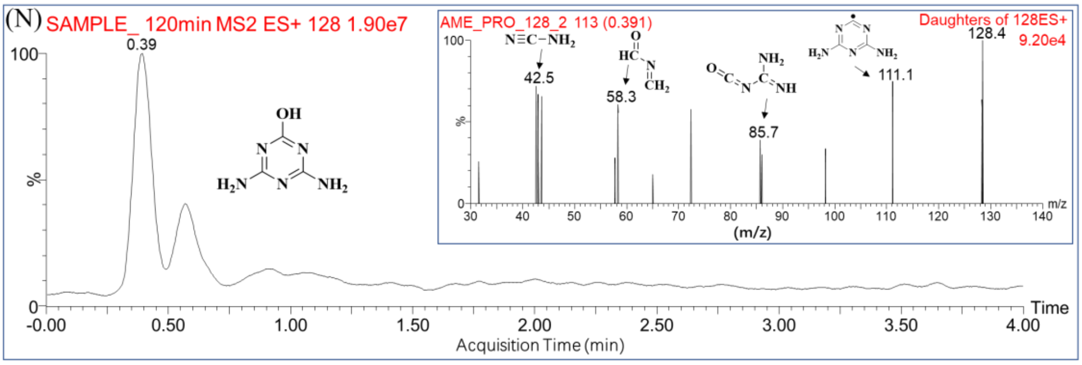
(N)
SAMPLE_ 120min MS2 ES+ 128 1.90e7
0.39
Acquisition Time (min)
Time
AME_PRO_128_2 113 (0.391)
Daughters of 128ES+
9.20e4
42.5
58.3
85.7
111.1
128.4
(m/z)

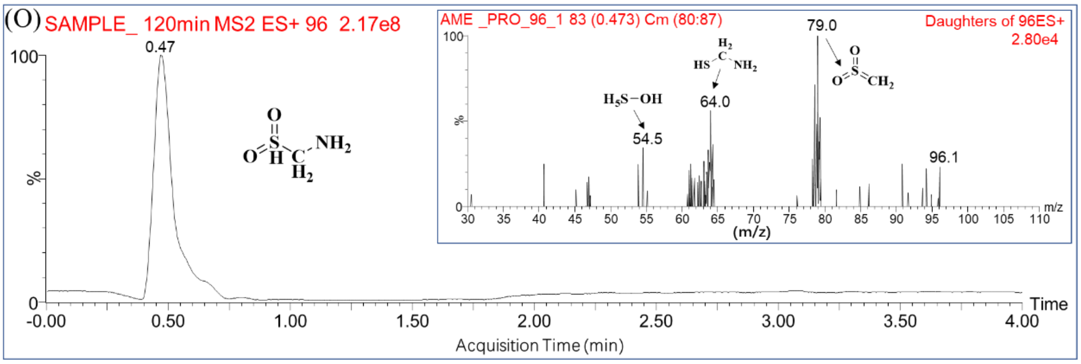
(O)
SAMPLE_ 120min MS2 ES+ 96 2.17e8
0.47
Acquisition Time (min)
Time
AME _PRO_96_1 83 (0.473) Cm (80:87)
Daughters of 96ES+
2.80e4
54.5
64.0
79.0
96.1
(m/z)

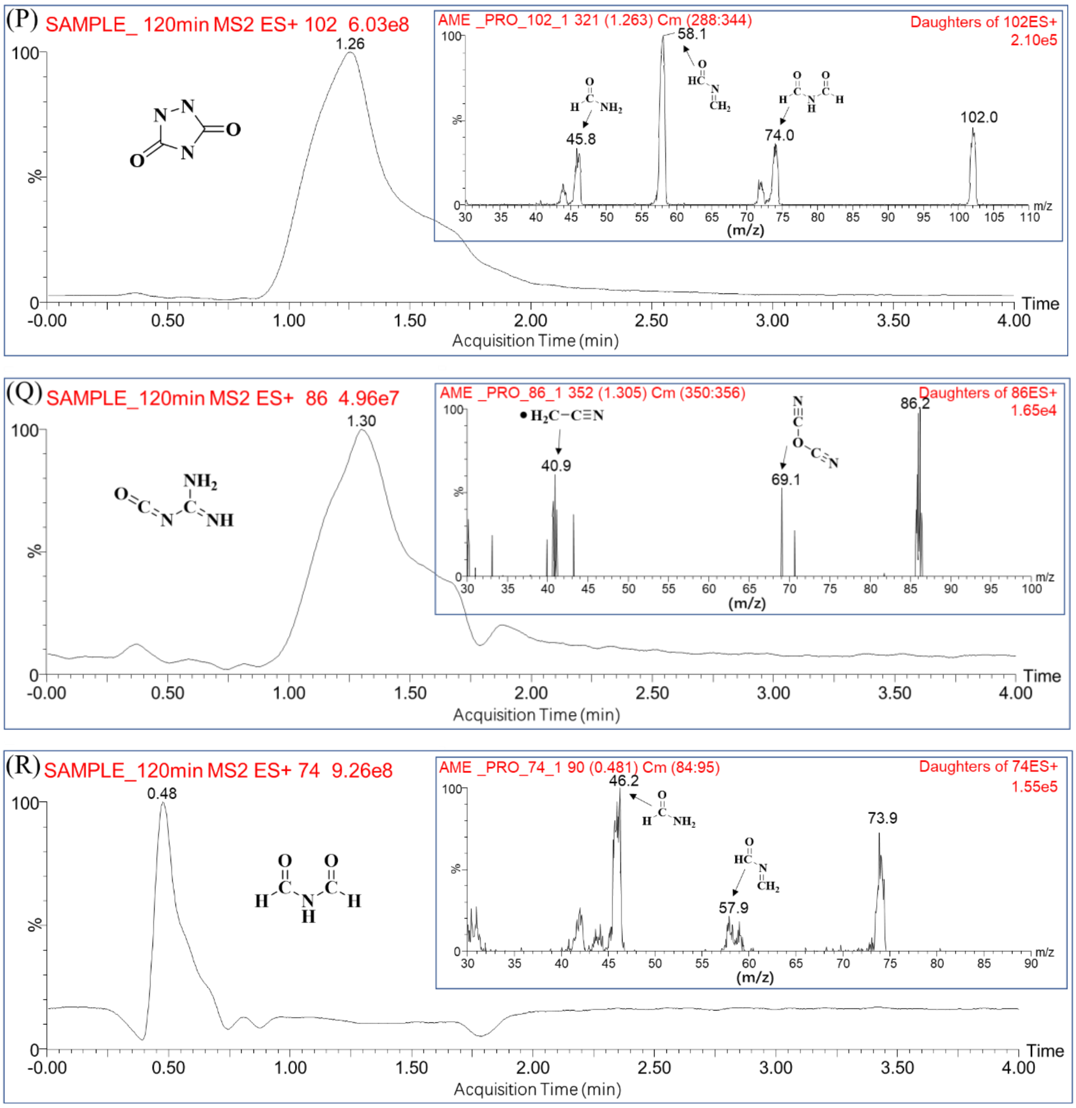


**Figure S7A-R.** Extract ion chromatography （EIC） analysis of the reaction intermediates for degradation of AME over 8Ppa/001T photocatalyst and the product ion scan spectrometry of identified reaction intermediates (inset).

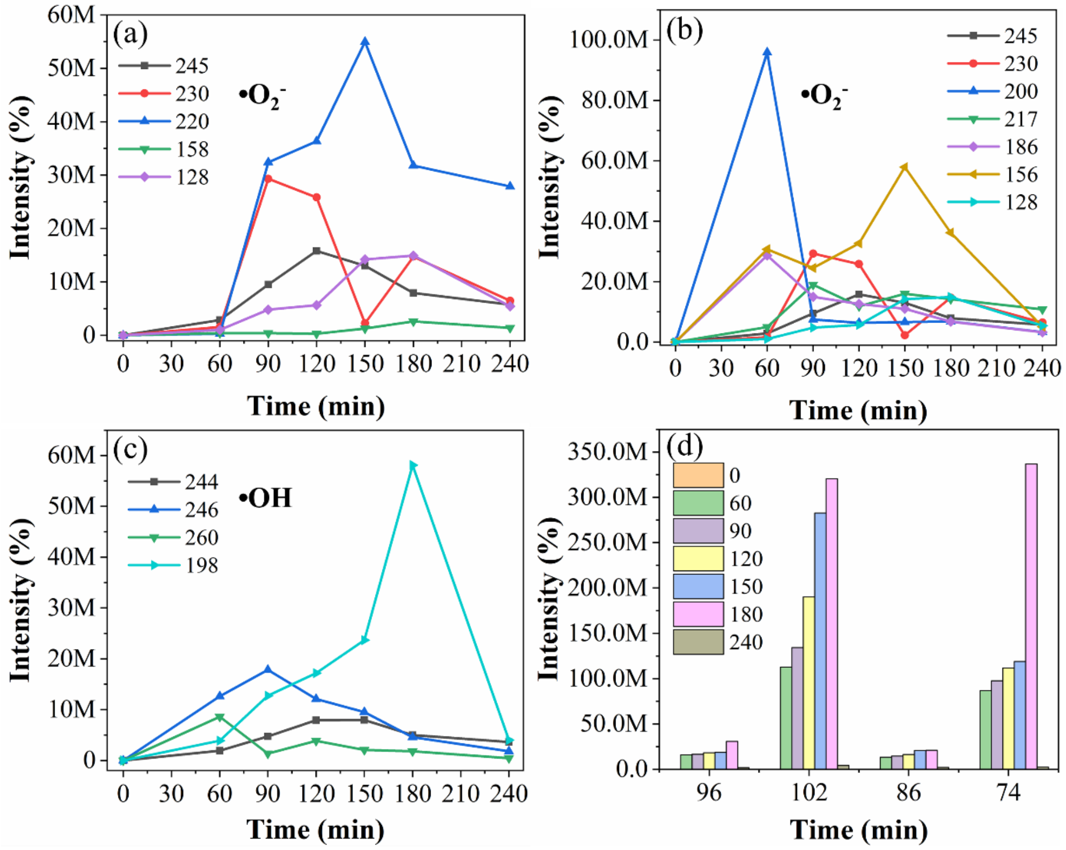


**Figure S8.** Fragment change trend chart. (a) Trends in the fragmentation of $\bullet O_2^-$ radical attack route I; (b) Trends in the fragmentation of $\bullet O_2^-$ radical attack route II;; (c) Trends in the fragmentation of •OH radical attack route and (d) Trend of fragments after opening the loop).

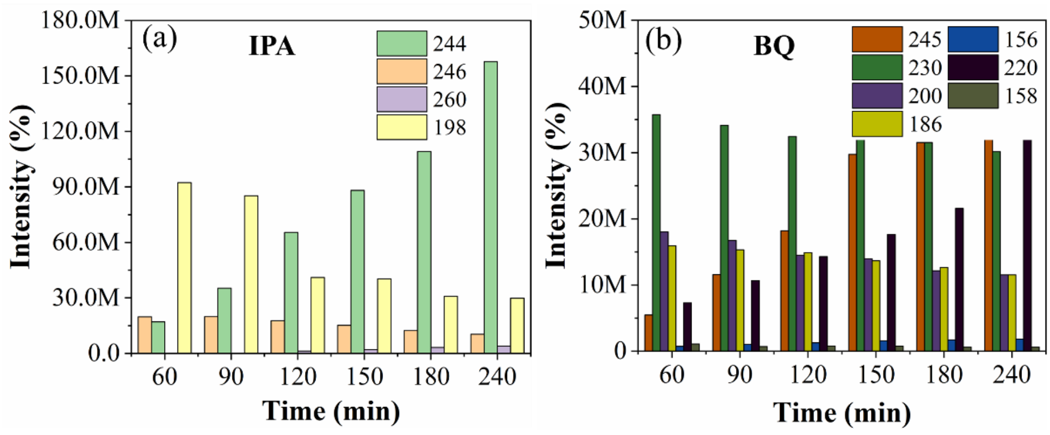


**Figure S9.** (a) Trends of individual ionic fragments after the addition of isopropanol scavengers; (b) Trends of individual ionic fragments after the addition of benzoquinone scavengers.

The overall fragment shows a tendency to delay or increase without decreasing at

the highest point, indicating that the scavenging agent captures the corresponding characteristic fragment and delays the reaction of the corresponding path.
